# Relationship between serum homocysteine, fibrinogen, lipoprotein-a level, and peripheral arterial disease: a dose–response meta-analysis

**DOI:** 10.1186/s40001-022-00870-1

**Published:** 2022-11-21

**Authors:** Hecheng Wang, Pengpeng Wu, Deying Jiang, Hao Zhang, Jian Zhang, Yu Zong, Yanshuo Han

**Affiliations:** 1 School of Life and Pharmaceutical Sciences, Dalian University of Technology, Dalian, China; 2 Department of Vascular Surgery, Dalian Municipal Central Hospital, Dalian, China; 3 Department of Vascular Surgery, The First Hospital of China Medical University, Shengyang, China

**Keywords:** Peripheral arterial disease, Homocysteine, Fibrinogen, Lipoprotein(a), Dose–response meta-analysis

## Abstract

**Aim:**

At present, the relationship between serum homocysteine (Hcy), fibrinogen (FIB), lipoprotein-a (LPa), and PAD is uncertain, and there has been no meta-analysis to establish the dose–response relationship between their exposure levels and PAD.

**Methods and results:**

Relevant literature published in PubMed, Embase, and Web of Science was retrieved. The robust error meta-regression method was used to assess the linear and non-linear dose–response relationship between exposure level and PAD risk. A total of 68 articles, involving 565,209 participants, were included. Combined with continuous variables, the serum Hcy, FIB, and LPa levels of PAD patients were significantly higher than those of healthy individuals. The odds ratios (ORs) of PAD for individuals with high Hcy, FIB, and LPa levels compared with those with low levels were 1.47, 1.14, and 1.76, respectively. The study also showed that circulating Hcy, FIB, and LPa were significantly elevated in patients with PAD compared with controls. The level of Hcy and the risk of PAD presented a U-shaped distribution. The nonlinear dose–response model showed that each 1 μmol/L increase in serum Hcy increased the risk of PAD by 7%. Similarly, for each 10 mg/dL FIB and 10 mg/dL LPa increases, the risk of PAD increased by 3% and 6%, respectively.

**Conclusions:**

This meta-analysis provided evidence that elevated Hcy, PIB, and LPa levels may increase the risk of PAD, and the risk of PAD increases with the increase in serum exposure within a certain range. By controlling Hcy level, the incidence of PAD may be reduced to control the PAD growing epidemic.

Trial registration number: PROSPERO (CRD42021250501), https://www.crd.york.ac.uk/prospero/

**Supplementary Information:**

The online version contains supplementary material available at 10.1186/s40001-022-00870-1.

## Introduction

Peripheral arterial disease (PAD) is characterized by atherosclerosis of the lower limbs. It is the third most common manifestation of atherosclerotic vascular disease after coronary artery disease (CAD) and stroke. PAD affects individuals all over the world [1, 2]. At present, the incidence of PAD is steadily increasing, accounting for 2–10% of the total population and even 20% of patients over 70 years [3, 4]. Although the diagnosis of the ankle/brachial index (ABI) is specific and patients with PAD have a high risk of cardiovascular events, PAD is still often undiagnosed or underestimated [5, 6]. The diagnosis of PAD heralds severe dysfunction, which is characterized by lower limb pain during exercise, called claudication, and severe limb ischemia and limb loss [7, 8]. Symptomatic PAD is usually characterized by intermittent claudication, adversely affecting patients’ quality of life and resulting in functional impairment [9]. Therefore, PAD results in a heavy burden and great pain in the affected families, patients, and even the society as a whole.

Although the etiology and pathogenesis remain unclear, PAD is considered a multifactorial disease; the pathophysiological factors causing peripheral arterial occlusion are complex, and atherosclerosis is the main pathophysiological basis of PAD [10]. The formation of atherosclerotic plaque is based on the gradual accumulation of lipids and inflammatory cells in the arterial wall [11, 12]. Several circulating biomarkers have been proposed to diagnose PAD, especially fibrinogen (FIB), homocysteine ​​(Hcy), lipoprotein a (LPa), C-reactive protein (CRP), D-dimer, and IL-6. Simple, inexpensive, and easy-to-detect markers of inflammation and thrombosis would play an important role in the positive diagnosis or risk stratification of PAD. However, there is no ideal and specific serum biomarker for clinical detection of PAD.

Reducing Hcy level may decrease the risk of PAD, and Hcy level has prognostic significance. However, the association between a mildly elevated Hcy level and risk of PAD is controversial. Although the relationship between circulating FIB and PAD has widely been reported in previous original studies and meta-analyses, inconsistent results have also been obtained in recent studies. For example, Small et al. determined hemostatic factors and their contribution to PAD; they suggested that circulating FIB level was not associated with PAD [13]. Several studies found that increased serum FIB concentration was associated with the presence of symptomatic PAD, independent of traditional and nontraditional cardiovascular risk factors [6, 14]. At present, the relationship between serum apolipoprotein-a (LPa) and PAD is still controversial, and there have been no meta-analyses to evaluate the dose–response relationship between these circulating biomarker levels and PAD.

Evaluating whether there is a dose–response relationship between certain exposure levels and disease outcomes is an important task of epidemiological research. It provides strong evidence for pathogenic inference and can promote the exploration of disease prevention and treatment measures. Therefore, this dose–response meta-analysis evaluated the relationship between serum FIB, Hcy, LPa and PAD, which may be of great significance for identifying the risk of PAD and improving the clinical course of PAD patients. We conducted this updated overall and dose–response meta-analysis to further explore the association between circulating biomarkers and risk of PAD.

## Methods

### Search strategy

This systematic review was registered at PROSPERO (www.crd.york.ac.uk/PROSPERO) as CRD CRD42021250501 on June 1, 2021. We carried out the meta-analysis in accordance with the Preferred Reporting Items for Systematic Reviews and Meta-analyses (PRISMA) guidelines [15]. The PubMed, Embase, and Web of Science databases were searched by two of the authors until April 20, 2021, without the restriction of language and publication date for eligible studies. Taking the relationship between serum Hcy and PAD as an example, the other two studies on the relationship between exposure levels and PAD can be deduced by analogy. We selected articles that reported on the relationship between homocysteine and PAD. Our search combined keywords and MeSH terms, and the search strategy for all databases is shown in Additional file 1: Table S1. In addition, reference lists of the retrieved articles and reviews on the subject were manually evaluated to identify any other relevant published articles. We did not include abstracts, grey literature, and unpublished studies.

### Eligibility criteria and study selection

The eligibility criteria were in accordance with the Population, Intervention/Exposure, Control, Outcomes, and Study design (PICOS) framework. Taking Hcy as an example, PICOS were included based on the following selection criteria:

- Population: Patients had to have an ankle–brachial index (ABI) value of ≤ 0.90 with intermittent claudication or asymptomatic PAD or chronic limb ischemia, and without diabetes, chronic renal failure, or metabolic syndrome.
- Intervention/Exposure: The level of fibrinogen had to be measured according to the Clauss method; the level of total homocysteine (free and protein bound) had to be determined by fluorescence polarization immunoassay; and Lp(a) concentration had to be determined by ELISA.
- Control: Healthy people had to be without PAD. Control individuals had be from the same geographic region, and the same exposure measurement methods had to exist between cases and controls.
- Outcomes: OR, mean ± SD.
- Study design: Cohort study, case–control study, and cross-sectional study.

Title/abstract screening: The study had to investigate the association between Hcy and PAD.

Full-text review: (1) the study had to be designed as an epidemiological study (e.g., cohort study, case–control study, or cross-sectional study); (2) the research had to report the Hcy levels of cases and controls and the standard deviation that we were able to estimate, or to report odds ratios (ORs), relative risks (RRs), or hazard ratios (HRs) with 95% confidence intervals (95% CIs) for Hcy and PAD; (3) patients had to have asymptomatic PAD, intermittent claudication, or chronic limb ischemia, and they did not have diabetes, chronic renal failure, or metabolic syndrome; (4) control individuals had to be healthy individuals without PAD from the same geographic region and matched with patients with respect to age, gender, and presence of type 2 diabetes mellitus.

Study selection was performed by two independent reviewers (H. W. and P. W.), and any discrepancies were resolved through discussion with a third reviewer (Y. H.).

### Data extraction and quality assessment

We extracted the following information from each study: author; country; year; ethnicity; study design; number of control individuals and PAD patients; gender ratio of controls and PAD patients; exposure level; ORs and 95% CIs for the highest vs the lowest level of the exposure variables; ORs and 95% CIs for different levels of the exposure variables; adjusting for confounders. As randomized control trials were not retrieved and only four articles from all the databases involved cross-sectional research, the Newcastle–Ottawa Scale (NOS) guidelines were used to evaluate the quality of the literature in this study. The NOS guidelines-modified studies that achieved six or more stars were considered to be of high quality, otherwise they were marked as low-quality studies.

### Statistical analysis

The studies including the serum levels of Hcy, FIB, and LPa of the PAD group and the control group were used to analyze the differences in serum Hcy, FIB, and LPa between patients with PAD and healthy individuals. We combined continuous variables into standard mean difference (SMD) and weighted mean difference (WMD). To find the relationship between the levels of exposures (Hcy, FIB, and LPa) and the risk of PAD, the summarized ORs and 95% CIs were assessed by random-effects models. Because of the low incidence of PAD (approximately 22.4/1000 person-years, with 95% CI of 20.8–24.0) [3], risk ratios were treated as ORs in most studies. We compared the ORs and 95% CIs of the highest level of exposures to the lowest level of exposures.

A potential linear and nonlinear dose–response relationship of Hcy, FIB, and LPa with the risk of PAD was examined by robust error meta-regression (REMR) approach described by Xu and co-workers, namely, we used inverse-variance weighted least squares (WLS) regression with cluster robust error variances [16] and we calculated study-specific slopes (linear trends) and standard error (SE) from the natural logarithms of the reported ORs and CIs across categories of anthropometric measures. The mean levels of Hcy, FIB, and LPa were assigned to the corresponding OR of each study; when these mean levels for this category were not reported, we calculated the average of the upper and the lower cutoff point to estimate the approximate midpoint [17]. When the highest or lowest category was open-ended, we assumed that the open-ended interval length was the same as the adjacent interval when estimating the midpoint [18].). In our data set, as the reference dose of exposures varied from study to study, the data first had to be centered [19]. Taking the average of the lowest dose of each study as the initial value of the exposure dose level, a restricted cubic spline model with knots was used to fit the potential nonlinear dose–response relationship. Thus, we calculated summary ORs and SEs for 1 μmol/L increase in Hcy, 10 mg/dL increase in FIB, and 10 mg/dL increase in LPa concentrations. Then, we run the same process without splines (using the linear dose fit) to calculate the linear trend.

Statistical heterogeneity was assessed using the *Q* test and *I*^2^; *P* < 0.1 and *I*^2^ > 50% indicated high heterogeneity between the studies. If there was a conflict between the *Q* statistic and the *I*^2^ statistic, the *I*^2^ statistic prevailed. Potential publication bias was assessed by Egger’s test and Begg’s test, with *P* < 0.05 indicating publication bias. To judge the robustness of the meta-analysis results, we carried out sensitivity analyses, including changing the effect model, trim-and-fill method, and analyses with excluding one study at a time.

## Results

### Study selection

**Fig. 1:**
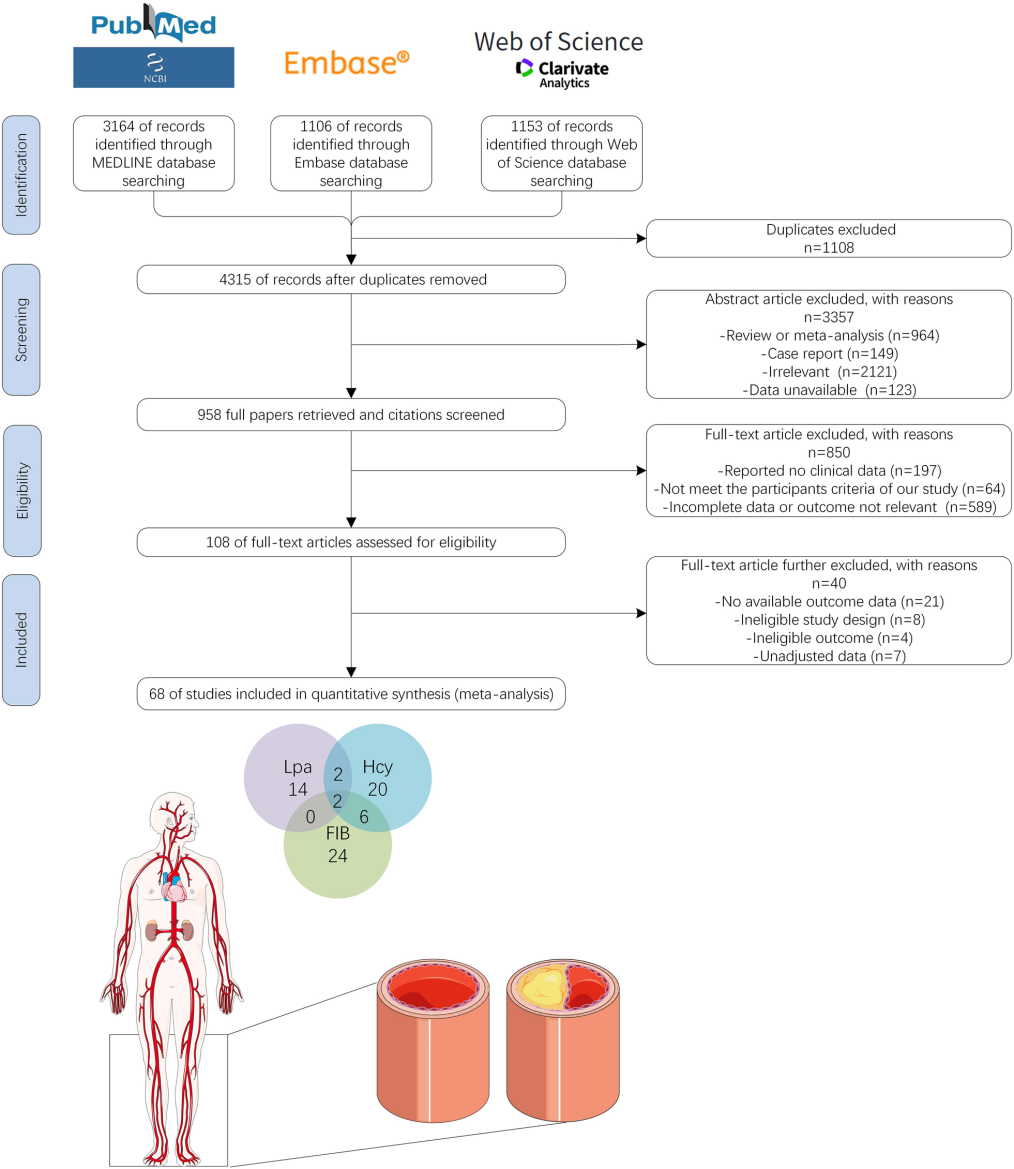
Flow chart for study inclusion and exclusion

The process of literature screening is presented in Fig. 1. Searching the databases (PubMed, Embase, and Web of Science), a total of 5423 literature records were obtained. After eliminating duplicate articles, the titles and abstracts of 4315 obtained articles were scanned. A total of 3357 references were excluded based on type, correlation, and duplication. Then, the remaining 958 articles were scanned in full text and screened in accordance with the inclusion and exclusion criteria mentioned above. Finally, the 108 articles that met the requirements were evaluated for quality. We excluded the articles with unusable outcome data, unqualified study design or outcome, and data that had not been adjusted for confounders. In the end, a total of 68 papers were included in this study, among which 30, 32, and 18 papers explored the relationships between serum Hcy, FIB, LPa and PAD, respectively. A total of 68 articles, involving 565,206 women and men, published between the year of 1989 and 2021, were included in this meta-analysis.

The detailed characteristics of the included studies are summarized in Table 1 and Additional file 2: Table S2. The quality assessment results are shown in Tables 2 and 3, and studies with more than six stars were considered high-quality studies.

**Table 1:** Demographic and clinical data of subjects enrolled in studies evaluating levels of Hcy, FIB and LPa in patients With PAD and control subjects

No.	Authors	Years	Study design	Country	Number of participants		Men n(%)		Age				Exposure	OR	SMD/WMD
					PAD	Controls	PAD	Controls	PAD		Controls				
									mean	SD	mean	SD			
1	Thomas Mueller [39]	2005	Case–control study	Austria	433	433	306 (70.7)	306 (70.7)	68	11.85	68	11.11	Hcy	✓	
2	Mengyuan Liu [36]	2020	Cohort study	China	3119	NG	NG	NG	NG	NG	NG	NG	Hcy	✓	
3	Dan Rong [37]	2017	Case–control study	China	240	240	203 (84.6)	195 (80.0)	63.6	12	59.2	7.8	Hcy	✓	✓
4	Janaka Weragoda [40]	2016	Case–control study	Sri Lanka	79	158	NG	NG	NG	NG	NG	NG	Hcy	✓	
5	Monica L. Bertoia [41]	2014	Cohort study	Netherlands	Women: 143 Men: 143	424 428	NG	NG	59.9 65.4	5.2 8.1	60 65.3	5.2 8.1	Hcy		
6	Tamam Mohamad [42]	2011	Cohort study	US	6590	NG	NG	NG	NG	NG	NG	NG	Hcy	✓	
7	Michelangelo Sartori [21]	2010	Cross-sectional study	Italy	CL: 181 CLI:110	210	118 (65.2) 69 (72.7)	122 (67.4)	68.7 72.2	9.4 9.4	68.6	5.8	FIB	✓	
8	Adriano Sabino [43]	2009	Case–control study	Brazil	44	37	26(59.1)	17 (45.9)	68.9	9.7	60.9	9.2	Hcy		✓
9	L. Garofolo [44]	2007	Cross-sectional study	Brazil	1008	NG	NG	NG	NG	NG	NG	NG	Hcy	✓	
10	Eliseo Guallar [45]	2005	Cross-sectional study	US	310	4137	139 (44.8)	2011 (48.6)	68.5	10.2	55.5	3.4	Hcy		
11	Matthew A. Allison [46]	2006	Cross-sectional study	US	275	6378	126 (45.8)	2981 (46.7)	70	9	62	10	FIB + Hcy		✓
12	P. Eller [20]	2005	Cross-sectional study	Austria	241	241	164 (68.0)	164 (68.0)	68.6	11.8	69.3	11.4	Hcy	✓	
13	T. Mueller [47]	2004	Case–control study	Austria	100	100	47 (47)	47 (47)	76	7.4	76	8.1	Hcy	✓	
14	Maurice A. A. J. van den Bosch [48]	2003	Case–control study	Netherlands	220	629	0	0	48	7	44.8	8.3	Hcy	✓	✓
15	DG.M. Bloemenkamp [49]	2002	Case–control study	Netherlands	212	475	0	0	48.2	7	45.5	8.1	Hcy	✓	
16	D. G. M. Bloemenkamp [50]	2002	Case–control study	Netherlands	150	412	0	0	48.7	6.9	45.5	7.9	Hcy		✓
17	H. Stricker [51]	2001	Case–control study	Switzerland	51	51	32 (62.7)	32 (62.7)	68.3	NG	68.5	NG	Hcy	✓	✓
18	Robert Loncar [52]	2001	Case–control study	Germany	40	40	NG	NG	51.8	7.5	45.6	6.8	Hcy		✓
19	Daniel Bunout [53]	2000	Case–control study	Chile	32	24	NG	NG	69.6	11	71.8	9.9	Hcy		✓
20	L. Todesco [54]	1999	Case–control study	Switzerland	63	106	29 (46.0)	58 (54.7)	74	10	76	16	Hcy		✓
21	Wibert S. Aronow [55]	1998	Cross-sectional study	US	men: 51 women: 96	107 266	NG	NG	82 84	9 8	80 81	8 8	Hcy		✓
22	M.R. Malinow [56]	1989	Case–control study	US	47	29	26 (55.3)	18 (62.1)	70.1	10.6	65.9	3.9	Hcy		✓
23	Lloyd M. Taylor [57]	1991	Case–control study	US	214	29	110 (51.4)	18 (62.1)	65	11.5	66	3.9	Hcy		✓
24	Aeron M. Small [13]	2020	Cohort study	US	24,009 5373 1925	150,983 42,485 18,285	23,416 (97.5%) 5166 (96.1%) 1885 (97.9%)	138,753 (91.9%) 36,188 (85.2%) 16,400 (89.7%)	74.4 69.6 71.6	9.4 9.4 9.6	66.9 60.4 59.0	13.2 11.6 14.8	FIB	✓	
25	Savas Celebi [58]	2020	Cross-sectional study	Turkey	152	128	126 (82.90)	86 (67.18)	69.01	11.13	58.13	12.78	FIB	✓	
26	Alexandr Ceasovschih [22]	2020	Case–control study	Romania	216	80	176 (81.5)	64 (80)	69	15	68	16	FIB	✓	
27	C. Roncal Mancho [59]	2014	Case–control study	Spain	88	20	NG	NG	71	11	80	5	FIB		✓
28	S. Marlene [60]	2014	Cohort study	US	113	727	113 (100)	727 (100)	68	10	67	11	FIB	✓	
29	Anetta Undas [61]	2010	Case–control study	Poland	106	106	82 (77.4)	79 (74.5)	57.1	6.9	56.4	6.8	FIB + Hcy		✓
30	Emile L.E. de Bruijne [62]	2010	Case–control study	Netherlands	47	141	13 (27.7)	39 (27.7)	43.2	7.9	43	7.6	FIB		✓
31	Laura M Reich [63]	2007	Cohort study	US	441	13,939	159 (36)	6273 (45)	57	7.4	54	7.4	FIB	✓	
32	Roberto Antonio Mangiafico [64]	2006	Case–control study	Italy	164	164	122 (74)	120 (73)	70	3.4	70.3	3.7	FIB		✓
33	E.A. Kaperonis [65]	2006	Case–control study	Greece	51	30	43 (84.3)	21 (70)	70	7.3	67	13.6	FIB		✓
34	Rachel P. Wildman [66]	2005	Cohort study	US	4787	NG	NG	NG	NG	NG	NG	NG	FIB	✓	
35	Khurram Nasir [67]	2005	Cross-sectional study	US	220	3729	72 (36)	1790 (48)	68	16.3	54	12.2	FIB	✓	
36	Elizabeth Selvin [6]	2004	Cross-sectional study	US	141	2033	65 (46.2)	980 (48.2)	68.7	1.5	55.7	0.4	FIB		✓
37	A. Kursat Bozkurt [68]	2004	Case–control study	Turkey	20	20	20 (100)	20 (100)	58.5	8.5	49.6	9.2	FIB		✓
38	Felicity B. Smith [69]	2003	Cross-sectional study	UK	104	663	56 (53.9)	317 (47.8)	65.8	5.1	63.7	5.1	FIB		✓
39	Andrew J. Makin [70]	2003	Case–control study	UK	234	50	145 (62)	27 (54)	68.6	10	68.6	10	FIB		✓
40	R. Giunta [71]	2001	Case–control study	Italy	27	20	15 (55.6)	11 (55.0)	66	11	65	10	FIB		✓
41	Wouter T. Meijer [14]	2000	Cohort study	Netherlands	men:2589 women:3861	NG	NG	NG	NG	NG	NG	NG	FIB + Hcy	✓	
42	Andrew Blann [72]	1998	Cross sectional study	UK	95	120	75 (79)	84 (70)	62	9	56	13	FIB		✓
43	Pavel Poredoš [73]	1996	Case–control study	Slovenia	33	19	12 (66.7); 9 (60.0)	16 (84.2)	63 ± 8.3 69 ± 10.4	NG	63	9.5	FIB		✓
44	Thomas Herren [74]	1994	Case–control study	Switzerland	22	13	14 (63.6)	9 (69.2)	64.8	8	63	7.5	FIB		✓
45	R. R. Fabsitz [75]	1998	Case–control study	US	women:145 men:81	2401 1597	NG	NG	60.3 59.6	7.9 8	56.1 55.5	7.8 8	FIB		✓
46	Agnieszka Okraska-Bylica [76]	2012	Case–control study	Poland	≤ 55: 31 > 55: 32	40	23 (74.2) 25 (78.1)	28 (70)	53 62	3 6.3	52.5	3.3	FIB		✓
47	Shuai Bing Li [77]	2013	Cross sectional study	China	145	837	69 (47.5)	251 (30)	62.67	11.12	61.27	10.59	Hcy FIB	✓	✓
48	G.C.Leng [78]	1995	Cross-sectional study	Scotland	131	722	63 (48.1)	377 (52.2)	66.8	5.7	63.6	5.4	FIB		✓
49	Pirjo Mustonen [79]	1998	Case–control study	Finland	15	15	10 (66.7)	10 (66.7)	59	8	57	11	FIB		✓
50	Azin Kheirkhah [80]	2020	Case–control study	Austria	248	251	NG	NG	58.3	6.3	56.9	9.5	LPa		✓
51	Hugh Tunstall-Pedoe [81]	2017	Cohort study	Scotland	499	NG	NG	NG					Lpa	✓	
52	N. Tmoyan [82]	2017	Case–control study	Russian	61	130	49 (80)	55 (42)	64.1	10.5	54.7	10.5	LPa	✓	✓
53	Nketi I. Forbang [38]	2016	Cohort study	US	4618	NG	NG	NG	NG	NG	NG	NG	LPa		
54	Anja Laschkolnig [83]	2014	Case–control study	Germany	241	246	241 (100)	246 (100)	58	6	56	9	LPa		✓
55	Zi Ye [84]	2012	Case–control study	US	211	NG	NG	NG	NG	NG	NG	NG	LPa + FIB	✓	
56	Monica L. Bertoia [85]	2013	Case–control study	Korea	women: 144 men: 143	432 429	NG	NG	59.9 65.4	5.2 8.1	60 65.3	5.2 8.1	LPa		✓
57	Annie M. Be´rard [86]	2013	Case–control study	French	113	241	89 (78.8)	186 (77.2)	39	7.8	33.1	6	Lpa + FIB + Hcy	✓	✓
58	Deepti Gurdasani [87]	2012	Cohort study	UK	596	212,385	NG	NG	NG	NG	NG	NG	LPa		
59	Stefano Volpato [88]	2010	Cohort study	Italy	1002	NG	NG	NG	NG	NG	NG	NG	LPa		
60	Aruna D. Pradhan [89]	2008	Cohort study	US	100	27,835	0	0	59.3	7.3	54.7	7.1	FIB Hcy LPa	✓	
61	Joachim H. Ix [90]	2008	Case–control study	US	104	164	49 (47)	77 (47)	69	10	68	9	Hcy + FIB		✓
62	Benjamin Dieplinger [91]	2007	Case–control study	Austria	213	213	158 (74)	158 (74)	66	10.37	66	10.37	LPa		✓
63	G.B. Vigna [92]	2006		Italy	67	NG	NG	NG	NG	NG	NG	NG	LPa	✓	
64	Francesco Sof [93]	2005	Case–control study	Italy	280	280	216 (77.1)	216 (77.1)	69	40.7	70	45.9	Hcy + LPa	✓	
65	Curt Diehm [94]	2004	Cross-sectional study	Germany	1230	NG	NG	NG	NG	NG	NG	NG	LPa	✓	
66	Paul M. Ridker [95]	2001	Case–control study	US	140	140	NG	NG	58	8.8	57.7	8.9	Hcy FIB	✓	
67	Kim Sutton-Tyrrell [96]	1995	Cohort study	US	369	NG	NG	NG	NG	NG	NG	NG	LPa	✓	
68	Mark Trinder [97]	2020	Cohort study	UK	2283	NG	NG	NG	NG	NG	NG	NG	LPa	✓

**Table 2:** Newcastle–Ottawa Scale of cohort studies

Study	Years	SELECTION				COMPARABILITY	OUTCOME		
		Representativeness of exposed cohort	Selection of the non‐exposed cohort	Ascertainment of exposure	Outcome of interest was not present	Comparability of exposure and non‐exposure	Ascertainment of outcome	Follow-up enough for outcome	Adequacy of follow-up
Small	2020	★	★	★	★	★★	★	★	★
Grenon	2014	★	★	★	★	★★	★	★	★
Reich	2007	★	★	★	★	★	★		
Wildman	2005	★	★	★	★	★★	★		
Smith	2003	★	★	★	★	★	★	★	★
Meijer	2000	★	★	★	★	★★	★		
LI	2013	★	★	★	★	★★	★		
Liu	2020	★	★	★	★	★★	★	★	★
Bertoia	2014	★	★	★	★	★★	★		
Mohamad	2011	★	★	★	★	★	★	★	
Garofolo	2007	★		★	★	★★	★		
Guallar	2005	★		★	★	★★	★		
Allison	2006	★	★	★	★	★★	★		★
ELLER	2005	★	★	★	★	★★	★		★
Aronow	1998	★	★	★	★	★★	★		
Pedoe	2017	★		★	★	★★	★	★	
Forbang	2016	★		★	★	★★	★		
Gurdasani	2012	★	★	★	★	★★	★	★	
Volpato	2010	★	★	★	★	★	★	★	★
Pradhan	2008	★		★	★	★★	★	★	★
Diehm	2004	★	★	★	★	★★	★		
Tyrrell	1995	★	★	★	★	★★	★		
Trinder	2020	★	★	★	★	★	★

**Table 3:** Newcastle–Ottawa Scale of case–control studies

Study	Years	Selection				Comparability	Exposure		
		Case definition adequate	Representativeness of the cases	Selection of controls	Definition of control	Comparability of cases and controls	Ascertainment of exposure	Same method of cases and controls	Non‐response rate
Ceasovschih	2020	☆	☆		☆	☆☆	☆	☆	
Celebi	2020	☆	☆	☆	☆	☆☆	☆	☆	☆
Mancho	2014	☆			☆	☆☆	☆	☆	
Undas	2010	☆	☆	☆	☆	☆	☆	☆	
Bruijne	2010	☆	☆	☆	☆	☆☆	☆	☆	
MANGIAFICO	2006	☆	☆	☆	☆	☆	☆	☆	
Kaperonis	2006	☆	☆	☆		☆	☆	☆	☆
Nasir	2005	☆			☆	☆	☆	☆	☆
Selvin	2004	☆		☆	☆	☆	☆	☆	
Bozkurt	2004	☆	☆	☆	☆	☆☆	☆	☆	
Makin	2003	☆	☆	☆	☆	☆	☆	☆	
Giunta	2001	☆	☆	☆	☆	☆☆	☆	☆	
Blann	1998	☆	☆	☆	☆	☆☆	☆	☆	
Mustonen	1998	☆	☆	☆	☆	☆☆	☆	☆	☆
Poredoš	1996	☆	☆	☆		☆	☆	☆	
LENG	1995	☆	☆	☆	☆	☆☆	☆	☆	☆
Herren	1994	☆	☆	☆	☆	☆☆		☆	
Fabsitz	1998	☆	☆	☆		☆	☆	☆	
Bylica	2012	☆	☆	☆	☆	☆☆	☆	☆	
Rong	2017	☆		☆	☆	☆	☆	☆	☆
Weragoda	2016	☆	☆	☆	☆	☆☆	☆	☆	☆
Sartori	2010	☆	☆	☆	☆	☆☆	☆	☆	
Sabino	2007	☆	☆	☆	☆	☆☆	☆	☆	
Mueller	2005	☆	☆	☆	☆	☆	☆	☆	☆
Mueller	2004	☆	☆	☆	☆	☆☆	☆	☆	
Bosch	2003	☆	☆	☆	☆	☆☆	☆	☆	
Bloemenkamp	2002	☆		☆	☆	☆☆	☆	☆	☆
Bloemenkamp	2002	☆		☆	☆	☆☆	☆	☆	☆
Stricker	2001	☆	☆	☆	☆	☆☆	☆	☆	
Loncar	2001	☆			☆	☆☆	☆	☆	
Bunout	2000	☆		☆	☆	☆☆	☆	☆	
Todesco	1999	☆	☆		☆	☆☆		☆	
Malinow	1989	☆			☆	☆☆	☆	☆	
Taylor	1991	☆	☆	☆	☆	☆	☆	☆	
Kheirkhah	2020	☆		☆	☆	☆☆	☆	☆	
Tmoyan	2017	☆		☆	☆	☆☆	☆	☆	
Laschkolnig	2014	☆	☆	☆	☆	☆	☆	☆	
Ye	2012	☆	☆	☆	☆	☆☆	☆	☆	
Bertoia	2013	☆	☆	☆	☆	☆☆	☆	☆	
Be´rard	2013	☆	☆	☆	☆	☆☆	☆	☆	☆
Ix	2008	☆	☆	☆	☆	☆☆	☆	☆	
Dieplinger	2007	☆	☆		☆	☆☆	☆	☆	☆
Vigna	2006	☆	☆	☆		☆	☆	☆	
Sof	2005	☆	☆	☆	☆	☆☆	☆	☆	☆
Ridker	2001	☆		☆	☆	☆☆	☆	☆	

### Association of Hcy, FIB, and LPa concentration with PAD

**Fig. 2:**
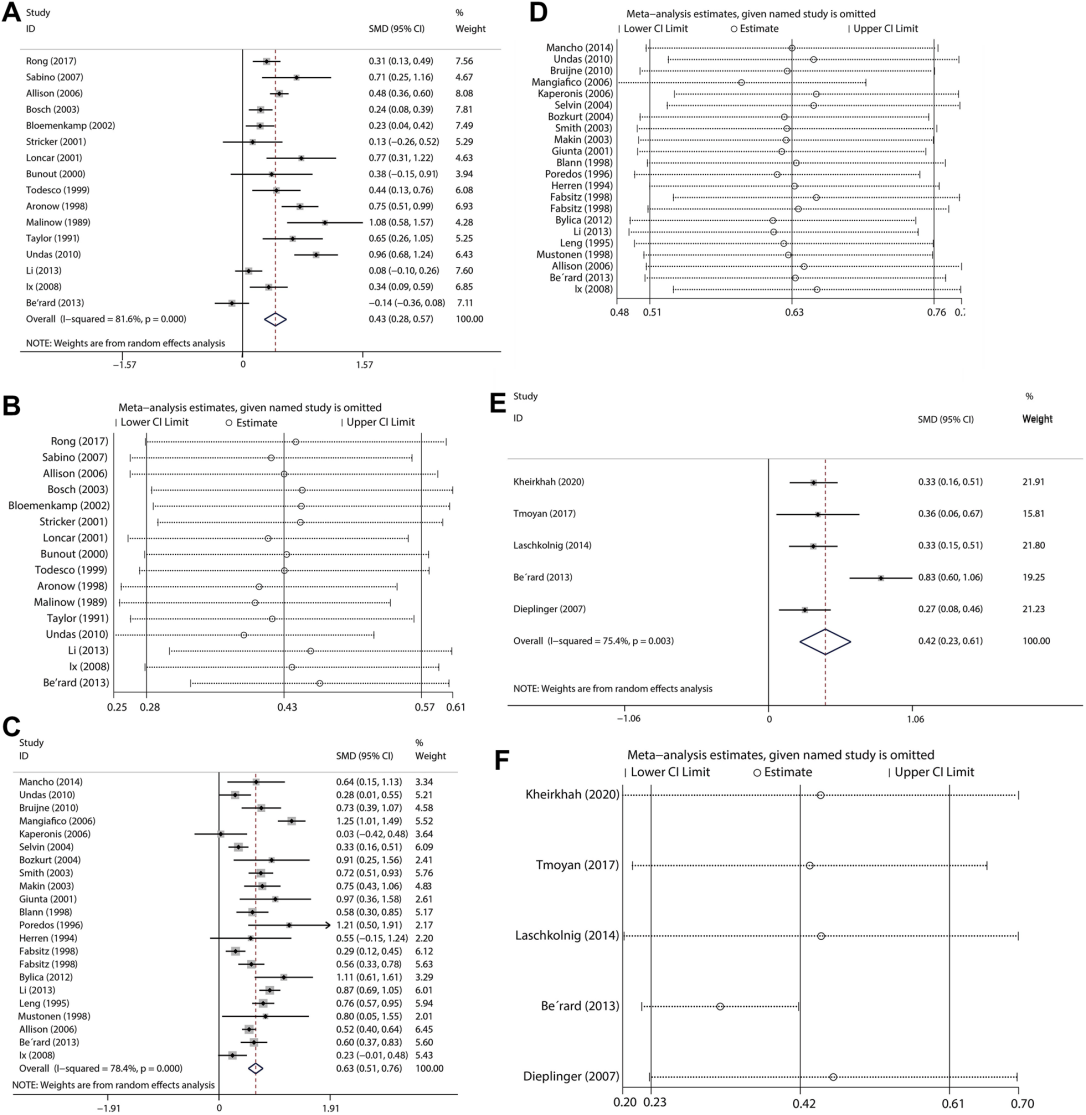
Circulating homocysteine levels **A** and trim-and-fill method analysis (**B**); fibrinogen level **C** and trim-and-fill method analysis (**D**); and lipoprotein-a level **E** and trim-and-fill method analysis **F** in patients with PAD and control subjects

Sixteen articles, with 11,687 participants, were included to compare the serum Hcy levels of PAD patients and healthy individuals. Using the random-effects model, the estimated value of the combined effect size of the SMD point was 0.429 (95% CI: 0.285–0.573, *I*^2^ = 81.6%, *P* < 0.001), i.e., the serum Hcy concentration of PAD patients was significantly higher than that of the controls (Fig. 2A). The point estimate of WMD was 2.252 (95% CI: 1.501–3.002), meaning that the serum Hcy concentration of PAD patients on average was 2.252 μmol/L higher than that of controls (Additional file 3: Fig. S1A). There was no indication of publication bias with Begg’s test (*P* = 0.096) and Egger’s (*P* = 0.207). Three studies were added with trim-and-fill method. The results did not change significantly, suggesting that the combined effect size results were robust. We also examined the impact of a single study on the results; the removal of any single study did not make a large change in the SMD, i.e., the result of the combined effect size was robust (Fig. 2B).

There were 21 studies, with 17,998 participants, exploring the difference in serum FIB level between patients with PAD and healthy individuals. The estimated value of the combined effect size of the SMD point was 0.631 (95% CI: 0.506–0.757, *I*^2^ = 78.4%, *P* < 0.001), indicating that the serum FIB concentration of PAD patients was significantly higher than that of the controls (Fig. 2C). The point estimate of WMD was 39.071 (95% CI: 30.120–48.022), indicating that the serum FIB concentration of PAD patients on average was 39.071 mg/dL higher than that of controls (Additional file 3: Fig. S1B). No significant publication bias was observed with Begg’s test (*P* = 0.284) and Egger’s test (*P* = 0.279). Four studies were added with trim-and-fill method; the SMD did not change significantly, suggesting that the combined effect size results were robust. Examination of the impact of a single study on the result revealed that the removal of any single study did not make a large change in the SMD (Fig. 2D).

Five studies, with 2,533 participants, were included to compare serum LPa levels between patients with PAD and controls. The estimated value of the combined effect size of the SMD point was 0.420 (95% CI: 0.231–0.609, *I*^2^ = 75.4%, *P* = 0.003), meaning that the serum LPa concentration of PAD patients was significantly higher than that of the controls (Fig. 2E). The point estimate of WMD was 39.071 (95% CI: 30.120–48.022), indicating that the serum LPa concentration of PAD patients on average was 39.071 mg/dL higher than that of controls (Additional file 3: Fig. S1C). No significant publication bias was found by Begg’s test (*P* = 0.806) and Egger’s (*P* = 0.503). Two studies were added with trim-and-fill method and the SMD did not change significantly, suggesting that the combined effect size results were robust. Examining the impact of a single study on the result revealed that the removal of any single study did not make a large change in the SMD (Fig. 2F).

### Elevated Hcy, FIB, Lpa, and risk of PAD

**Fig. 3:**
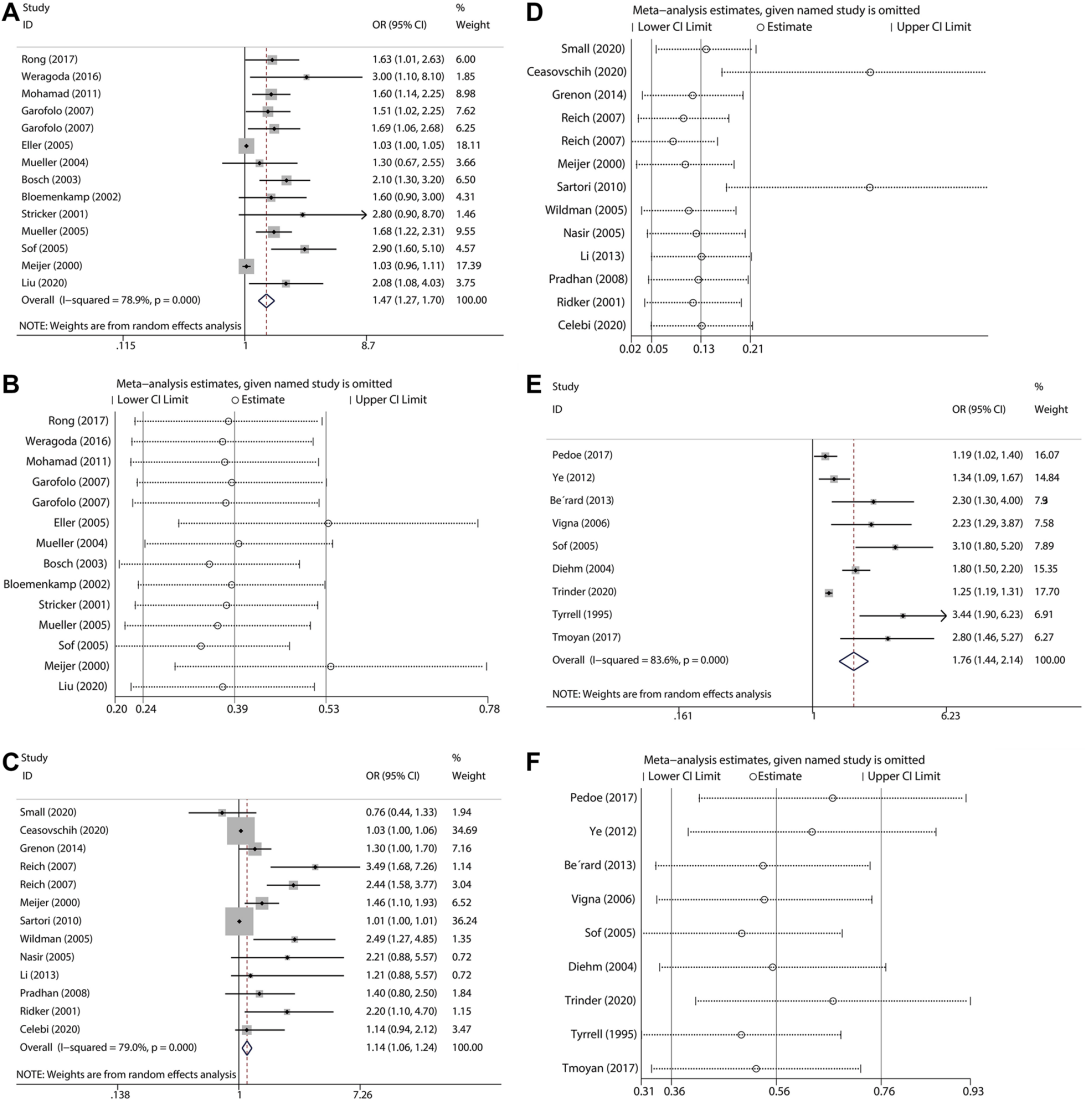
Prevalence of PAD in patients with homocysteine levels homocysteine ≥ 10 μmol/L vs those with less than 10 μmol/L **A** and two studies were added with trim-and-fill method **B** for homocysteine levels and PAD. Distribution of fibrinogen levels upper and lower status in controls and PAD patients and odds ratios (**C**), and trim-and-fill method analysis (**D**). Prevalence of PAD in patients with upper lipoprotein-a levels vs those with lower lipoprotein-a levels **E** and trim-and-fill method analysis (**F**)

Data extracted from 13 articles (21,630 participants) that compared the relative risk of PAD between individuals at the top level of Hcy and those at the bottom level of Hcy yielded a summary OR of 1.470 (95% CI: 1.274–1.696, *I*^2^ = 78.9%, *P* < 0.001; in Fig. 3A). No significant publication bias was found by Begg’s test (*P* = 0.913). We examined the impact of a single study on the results; removing two studies [14, 20] made the result exceed the confidence interval, but it did not reverse the result, and removing other studies did not change the results significantly (Fig. 3B).

Twelve studies, including 303,710 participants, were used to evaluate the risk of PAD in individuals with the highest FIB levels compared with those with the lowest FIB levels. The summary OR was 1.142 (95% CI: 1.005–1.237, *I*^2^ = 79.0%, *P* < 0.001; in Fig. 3C). There was no indication of publication bias with Begg’s test (*P* = 0.541). We examined the impact of a single study on the results; removal of two studies [21, 22] made the result larger than the confidence interval, but it did not reverse the result, and removing other studies did not change the results significantly (Fig. 3D).

Nine studies, including 5,764 participants, were used to evaluate the risk of PAD in individuals with high FIB levels; the summary OR was 1.755 (95% CI: 1.438–2.143, *I*^2^ = 83.6%, *P* < 0.001; in Fig. 3E). There was no evidence of publication bias with Begg’s test (*P* = 0.118). The result of the combined effect size was robust, because there was no significant change in the result after applying the trim-and-fill method, and the removal of any single study did not significantly change the result of the combined effect size (Fig. 3F).

### Dose–response analysis

**Fig. 4:**
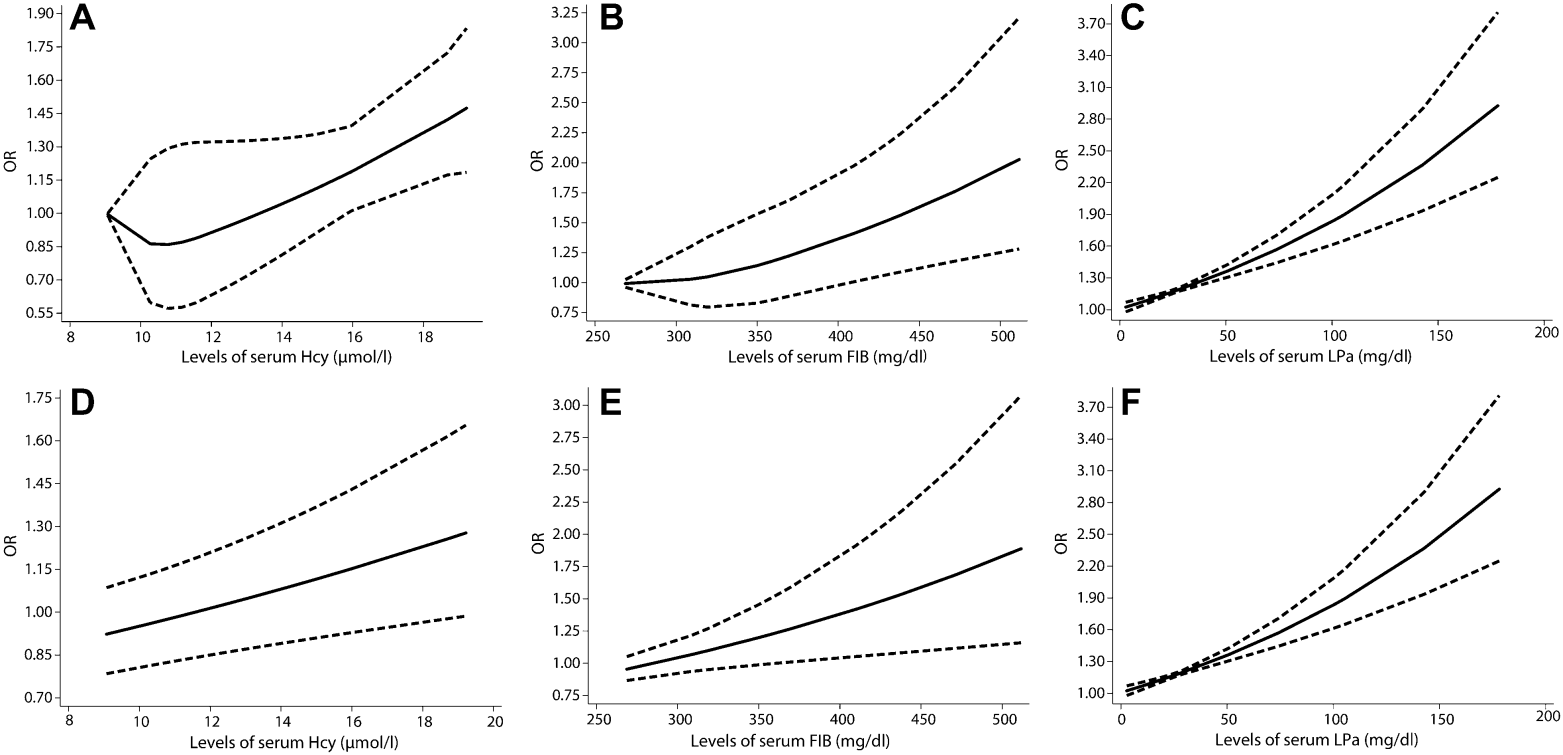
Linear and nonlinear dose–response analyses of circulating Hcy levels, FIB level and LPa level and risk of AAA. The nonlinear dose–response analysis of homocysteine, per 1 μmol/L (**A**), fibrinogen level, per 10 mg/dL (**B**), and lipoprotein-a level, per 1 mg/dL **C** and risk of PAD. The linear dose–response analysis of homocysteine, per 1 μmol/L (**D**), fibrinogen level, per 10 mg/dL (**E**), and lipoprotein-a level, per 1 mg/dL **F** and risk of PAD

Six studies (34,898 participants) were included to analyze the dose–response relationship between Hcy and PAD. The data were fitted using an RCS with three knots (at 7, 9, and 12) allowing for a potential nonlinear relationship. The regression parameter estimates of the first spline and the second spline were − 0.4353 (β1) and 0.4180 (β2), respectively (Table 4, Additional file 4: Table S3 and Additional file 5: Table S4). An increase in the Hcy concentration of 1 μmol/L resulted in a 7% increase in the risk of PAD (*P* = 0.398). The level of Hcy and the risk of PAD presented a U-shaped distribution (Fig. 4A). When the concentration of Hcy was higher than 11.7 μmol/L, the risk of PAD increased sharply. We used the same process to calculate the linear trend over the entire range of doses, and the risk of PAD increased 3% per each 1 μmol/L increase of Hcy concentration (Fig. 4B).

**Table 4:** Estimated regression parameters and standard errors by REMR model

	Hcy and PAD	FIB and PAD	Lpa and PAD
Knots	7, 9, 12	268, 318, 370	2, 19, 74
β1(SE)	− 0.4353 (0.4765)	0.0005 (0.0036)	0.0074 (0.0018)
β2(SE)	0.4180 (0.4254)	0.0020 (0.0028)	− 0.0026 (0.0036)
P for nonlinearity	0.398	0.882	0.722
β1(nonspline model)	0.0320	0.0028	0.0060

Five studies (37,933 participants) were used to analyze the dose–response relationship between FIB and PAD. An RCS was created (with three knots at 268, 318, and 370 of the dose distribution), which generated two splines, and these were then employed for the potential nonlinear dose-specific modeling. The regression parameter estimates of the first spline and the second spline were 0.0005 (β1) and 0.0020 (β2), respectively (Table 4, Additional file 4: Table S3 and Additional file 5: Table S4). The risk of PAD increased by 3% for each 10 mg/dL increase of FIB concentration (*P* = 0.882). Nonlinear dose–response analysis showed that the risk of PAD continued to increase as FIB increased (Fig. 4C). However, the nonlinear dose–response relationship also showed a flat curve over the typical range of FIB concentrations, suggesting that higher risks were associated with higher concentrations. Under the linear model, the risk of PAD increased by 3% for the same increment (Fig. 4D).

Five studies (247,709 participants) were included to analyze the dose–response relationship between LPa and PAD. There were three knots (at 2, 19, and 74 across the reported dose distribution) using RCSs. Estimated regression parameters were 0.007384 for β1 and − 0.002629 for β2 (Table 4, Additional file 4: Table S3 and Additional file 5: Table S4). An LPa increment of 1 mg/dL resulted in a 6% increase in the risk of PAD. Nonlinear dose–response analysis (Fig. 4E) showed a consistently increasing risk with increased LPa. Given the linear relationship (Fig. 4F), the linear trend (for 10 mg/dL increase) was 1.06, that is, the risk of PAD increased by 6% for each 10 mg/dL increase in LPa concentration.

### Subgroup analysis

**Fig. 5:**
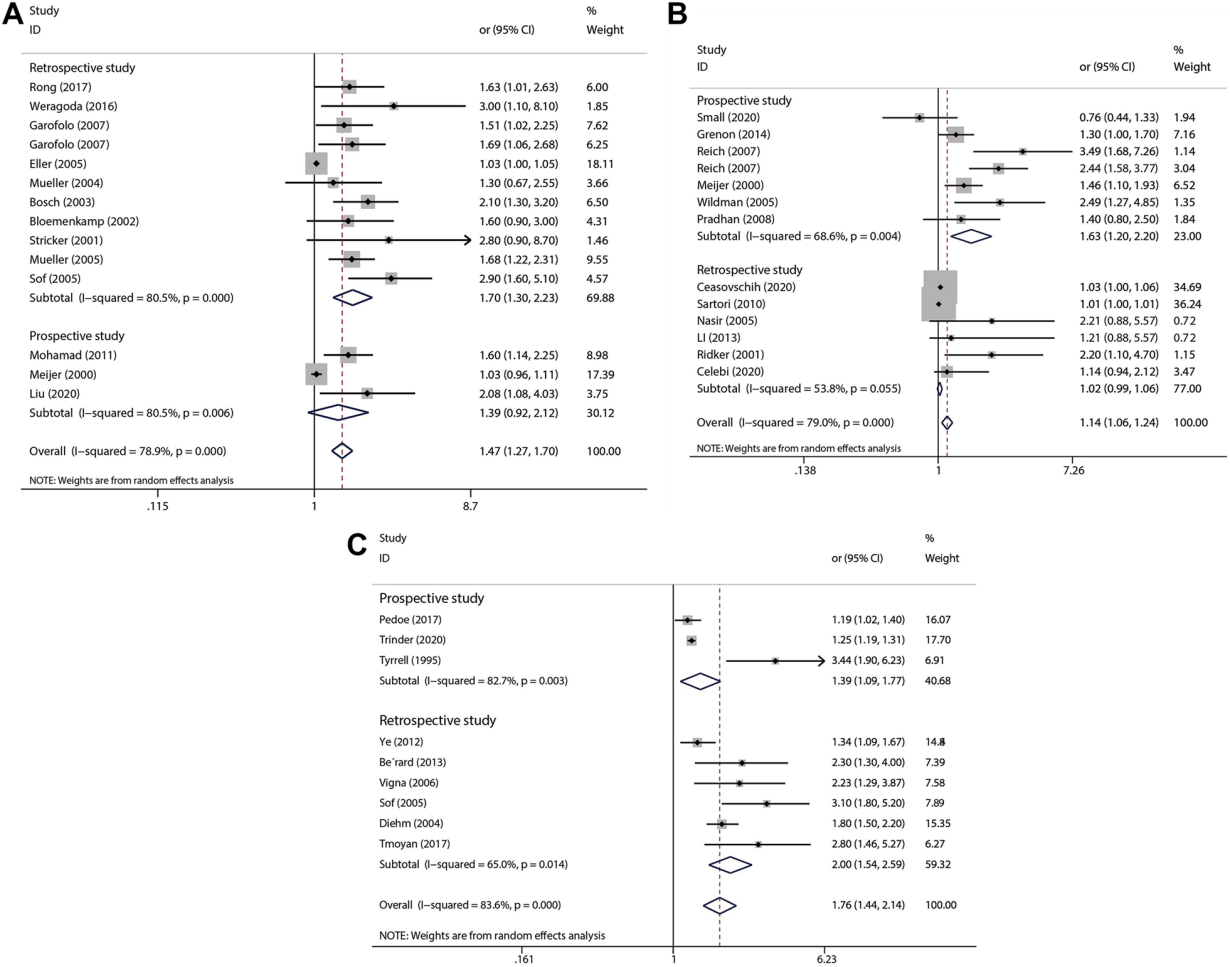
Subgroup analysis forest plot shows the OR and 95% CI for the association between circulating Hcy levels (**A**), FIB level **B** and LPa level **C** and risk of PAD

Subgroup analysis was performed based on study design, and studies were divided into prospective and retrospective studies. Retrospective studies include case–control studies and cross-sectional studies, while prospective studies are cohort studies. Due to limitations in the data included in the study, we performed subgroup analyses only to assess the risk between Hcy, FIB, and LPa and PAD. As shown in Fig. 5A, whether it is a combination of prospective studies or retrospective studies, the results show that HCY is a risk factor for PAD. When evaluating the relationship between FIB and PAD risk, combined prospective research results show that FIB is a risk factor for PAD, while combined retrospective studies have no effective results, as shown in Fig. 5B. As for the relationship between LPa and PAD risk, both prospective studies and retrospective studies have shown that LPa is a risk factor for PAD, as shown in Fig. 5C.

## Discussion

PAD commonly results from progressive narrowing of arteries in the lower extremities due to atherosclerosis. Previous studies have shown that PAD is associated with a significantly elevated risk of cardiovascular disease morbidity and mortality [23, 24]. PAD is also a common macrovascular complication of T2DM, which not only may contribute to initiation and aggravation of diabetic foot ulcer but is also an efficient predictor of cardiovascular mortality and morbidity.

### Main implications

In the present study, we found that the serum homocysteine levels of PAD patients were significantly higher than those of healthy individuals. In addition, the risk of PAD in individuals with high serum homocysteine was 1.47 times higher than that of the corresponding low-level population. Meanwhile, Hcy was significantly higher (pooled mean difference 2.25 μmol/L; 95% CI: 1.50–3.00, *P* < 0.0001) in patients with PAD compared with controls. Previously, a meta-analysis of 14 relevant studies showed that Hcy was significantly elevated (pooled mean difference + 4.31 μmol/L; 95% CI: 1.71–6.31, *P* < 0.0001 with significant heterogeneity) in patients with PAD compared with controls [25]. However, that study only reported the pooled relative risk of PAD when comparing the highest Hcy category group with the lowest Hcy category group and failed to explore the quantitative dose–response association between Hcy levels and risk of PAD. The present study is the first systematic dose–response meta-analysis of serum homocysteine levels and the risk of PAD. Regarding the nonlinear dose–response relationship between serum Hcy levels and PAD, we found that when the concentration of Hcy was higher than 11.7 μmol/L, every increase of 1 μmol/L in serum concentration of Hcy increased the risk of PAD by 7%. More importantly, we found that the relationship between serum Hcy level and the risk of PAD showed a *U*-shaped curve distribution. When the Hcy concentration was lower than 11.7 μmol/L, Hcy was not considered a risk factor for PAD; in contrast, when the Hcy concentration was higher than 11.7 μmol/L, the risk of PAD increased sharply with the increase of Hcy level. This conclusion is supported by the previously reported relationship between PAD and Hcy. [26]. Our findings may have important implications for public health. Elevated Hcy level may be a modifiable risk factor for PAD.

According to the results of our meta-analysis, controlling and maintaining a healthy fibrinogen level should be recommended to benefit health. Here, the risk of PAD in individuals with high serum fibrinogen levels was 1.14 times higher than that of the corresponding low-level population. Kremers et al. identified fibrinogen as a promising biomarker that represents different pathophysiological processes implicated in lower extremity PAD; in that meta-analysis, increased fibrinogen levels have been associated with an increased relative risk of mortality of 2.08 [27]. However, the study designs included in that meta-analysis were inconsistent, including prospective nested case–control studies, prospective cohort studies, and case–control studies. Thus, here we performed a meta-analysis focusing only on prospective cohort studies to investigate the dose–response association between the FIB level and risk of PAD. We also found that the relationship between serum FIB levels and the risk of PAD presented an approximately J-shaped curve distribution. When the FIB concentration was higher than 319.7 mg/dL, every 10 mg/dL increase in serum FIB concentration increased the risk of PAD by 3%. When the FIB concentration was lower than 319.7 mg/dL, the risk of PAD did not change significantly with the increase of FIB level; in contrast, when the FIB concentration was higher than 319.7 mg/dL, the risk of PAD increased sharply with the increase in FIB level.

We found that the serum lipoprotein-a (LPa) levels of PAD patients were significantly higher than those of healthy individuals. In addition, the risk of PAD in individuals with high serum LPa levels was 1.76 times higher than that of the corresponding low-level population. The dose–response relationship between serum LPa level and PAD indicated that as the LPa level increased, the risk of PAD increased. In addition, every 10 mg/dL increase in serum LPa concentration increased the risk of PAD by 6%.

### Potential mechanism

The pathogenesis of PAD is complex, involving a variety of genetic and environmental factors related to atherosclerosis and thrombosis, and the interaction between them [28]. The most common cause of PAD is atherosclerosis [29]. Atherosclerotic plaque causes narrowing or occlusion of the arteries, thereby reducing blood flow to the affected limb [29]. Thrombin-mediated conversion of plasma fibrinogen to fibrin, forming a relatively insoluble clot, is the final step in the clotting cascade. Fibrin has been shown to be a stable component of atherosclerotic plaque and to promote its growth [30]. In addition, FIB is also a determinant of inflammation biomarkers, acute phase reactants, and blood viscosity. Hyperhomocysteinemia has been reported to reduce thrombus permeability and solubility sensitivity, and the current study confirmed that Hcy is associated with certain fibrin clot markers in patients with PAD, despite a number of potential confounding factors [31]. In addition, Hcy is involved in various pathological processes, such as endothelial dysfunction, oxidative stress, and vascular remodeling, which further aggravate the impairment of vasodilation in PAD patients during exercise, resulting in motor dysfunction [32]. These mechanisms may explain how Hcy increases the risk of PAD. LPa is the main carrier of oxidized phospholipids in plasma, and it induces the activation of monocytes. The increased migration of these monocytes between endothelial cells leads to the production of proinflammatory cytokines and other cellular effects that contribute to the progression of atherosclerotic disease [33–35]. At present, the pathophysiological effects of serum Hcy, FIB, LPa, and PAD are still not fully understood, and more studies on the correlation mechanism are needed in the future.

### Advantages and limitations

Our meta-analysis has several strengths. To our knowledge, this is the first systematic dose–response meta-analysis of serum exposure levels of Hcy, FIB, and LPa and the risk of PAD, and the results obtained have a mutual collaborative relationship with previous research conclusions. Compared with the sample size limit of a single study, this meta-analysis included a total of 68 articles, with a total sample size of 565,209 patients, including > 56,754 PAD cases. The total sample size of the studies on the relationship between Hcy, FIB, LPa and PAD reached 65,886, 320,756, and 255,456, respectively. A large number of cases allowed us to determine the relationship between exposure levels and PAD risk. In addition, the 68 included articles were of high quality (all studies scored ≥ 6 stars). The method used in this study was the REMR method. Compared with previous studies using Generalized Least Squares (GLST) as a dose–response meta-analysis method, the REMR method eliminated the bias generated by the GLST method using the non-intercept model, resulting in better error estimation and a better visual fit to the data [16]. Moreover, our meta-analysis included prospective cohort studies, which may effectively avoid the possibility of a reverse relation and enhance the possibility of an etiological hypothesis. Finally, all the included studies have a relatively high quality, and the main results were robust after sensitivity analyses and Egger’s test.

Several potential limitations should be mentioned in this meta-analysis. First, according to previous reports, the relationship between exposure levels and the risk of PAD may vary slightly by gender and region [36–38], but we did not perform the subgroup analysis based on sex and region due to the limited data. Second, although the included studies adjusted for potential risk factors of PAD, residual confounders might exist because of the observational nature of the data. In addition, there was a large heterogeneity among the included studies, but the results of the sensitivity analysis showed that the combined effect size results were robust. Of course, the three exposure factors in this study are inherently controversial as risk factors for PAD, which may have led to high heterogeneity between the studies. Moreover, differences in the measurement of exposure levels may have also increased the heterogeneity. However, overall, the results of this study were stable, and the direction of exposure factors on PAD had never changed.

## Conclusions

Based on the above results, it can be reasonably concluded that serum Hcy, FIB, and LPa are related to the risk of PAD, and within a certain range of their serum levels, the risk of PAD increases with the increase in the serum level. These three exposure factors are expected to become serum biomarkers of PAD. Individuals with high serum Hcy, FIB, and LPa levels should be highly concerned about the risk of PAD, and early screening and appropriate treatment are crucial. By controlling the Hcy level, the incidence of PAD may be reduced to control the growing epidemic. Therefore, our research is of great significance for the prevention of PAD and for improving the diagnosis of the disease. Certainly, further observational studies with large sample sizes are needed to verify our results, and more studies are also needed to explore the underlying physiological and pathological mechanisms.

## Supplementary Information

**Additional file 1: Table S1.** Search strategy.

**Additional file 2: Table S2.** Characteristics of individual studies on Hcy, FIB, LPa and risk of PADs.

**Additional file 3: Figure S1**. Weighted mean difference (WMDs) and 95% confidence intervals (CIs) of the selected studies and the pooled Hcy levels (**A**), FIB level (**B**) and LPa level **C** in patients with PAD and control subjects.

**Additional file 4: Table S3.** Table of relative risks (95% confidence intervals) from the nonlinear dose–response analysis of Hcy, FIB, LPa and risk of PAD.

**Additional file 5: Table S4.** Table of relative risks (95% confidence intervals) from the linear dose–response analysis of Hcy, FIB, LPa and risk of PAD.

## Data Availability

The data sets used or analyzed during the current study are available from the corresponding author on request.

## Abbreviations

PAD: Peripheral artery disease
Hcy: Homocysteine
FIB: Fibrinogen
LPa: Lipoprotein(a)
OR: Odds ratio
REMR: Robust error meta-regression
SD: Standard error
CI: Confidence interval
CRP: C-reactive protein
CVD: Cardiovascular disease
DM: Diabetes mellitus
ABI: Ankle brachial index
BMI: Body mass index
SBP: Systolic blood pressure

## Code availability

Microsoft Excel 2010; Microsoft, Redmond, WA, USA. STATA meta.ado module (version 15.1; COLLEGE Station, TX, USA). This paper is not based on a previous communication to a society or meeting.
