# Supplementary material for: Relationship between serum homocysteine, fibrinogen, lipoprotein-a level, and peripheral arterial disease: a dose–response meta-analysis
Source: Eur J Med Res. 2022 Nov 21;27:261. doi: 10.1186/s40001-022-00870-1 (PMC9677707; doi:10.1186/s40001-022-00870-1)
Supplement: Supplementary file 1 — Additional file 1: Table S1. Search strategy. [file 40001_2022_870_MOESM1_ESM.docx]

**Supplementary File 1.** Search strategy.

Studies were selected that reported on relationship between serum Hcy, FIB, LPa and peripheral arterial disease (PAD). Search combined keywords and MeSH terms, and the search strategy for all literature database was as follow:

- "homocysteine"[Mesh] or 2-amino-4-mercaptobutyric acid or 2 amino 4 mercaptobutyric acid or Homocysteine, L-Isomer or Homocysteine, L Isomer or L-Isomer Homocysteine and "Peripheral Arterial Disease"[Mesh] or Arterial Disease, Peripheral or Arterial Diseases, Peripheral or Disease, Peripheral Arterial or Diseases, Peripheral Arterial or Peripheral Arterial Diseases or Peripheral Artery Disease or Artery Disease, Peripheral or Artery Diseases, Peripheral or Disease, Peripheral Artery or Diseases, Peripheral Artery or Peripheral Artery Diseases, which is for the relationship between serum Hcy and PAD;
- "Lipoprotein(a)"[Mesh] or Lipoprotein Lp(a) or Lipoprotein (a) or Lipoprotein a or Lipoprotein (a-) and "Peripheral Arterial Disease"[Mesh] or Arterial Disease, Peripheral or Arterial Diseases, Peripheral or Disease, Peripheral Arterial or Diseases, Peripheral Arterial or Peripheral Arterial Diseases or Peripheral Artery Disease or Artery Disease, Peripheral or Artery Diseases, Peripheral or Disease, Peripheral Artery or Diseases, Peripheral Artery or Peripheral Artery Diseases; which is for the relationship between serum LPa and PAD;
- "Fibrinogen"[Mesh] or Blood Coagulation Factor I or Coagulation Factor I or Factor I, Coagulation or Factor I or gamma-Fibrinogen or gamma Fibrinogen and "Peripheral Arterial Disease"[Mesh] or Arterial Disease, Peripheral or Arterial Diseases, Peripheral or Disease, Peripheral Arterial or Diseases, Peripheral Arterial or Peripheral Arterial Diseases or Peripheral Artery Disease or Artery Disease, Peripheral or Artery Diseases, Peripheral or Disease, Peripheral Artery or Diseases, Peripheral Artery or Peripheral Artery Diseases, which is for the relationship between serum FIB and PAD.
