# Supplementary material for: Relationship between serum homocysteine, fibrinogen, lipoprotein-a level, and peripheral arterial disease: a dose–response meta-analysis
Source: Eur J Med Res. 2022 Nov 21;27:261. doi: 10.1186/s40001-022-00870-1 (PMC9677707; doi:10.1186/s40001-022-00870-1)
Supplement: Supplementary file 2 — Additional file 2: Table S2. Characteristics of individual studies on Hcy, FIB, LPa and risk of PADs. [file 40001_2022_870_MOESM2_ESM.docx]

**Supplementary Table 2.** Characteristics of individual studies on Hcy, FIB, LPa and risk of PAD.

| Author | Year | Study design | Ethnicity | Number of participants | | Exposure | Description of quantiles of exposure | Relative effect (95% CI) | | Adjustment for  confounders |  |
| --- | --- | --- | --- | --- | --- | --- | --- | --- | --- | --- | --- |
|  |  |  |  | PAD | Controls |  |  | Univariate regression model | Multivariate regression model |  |  |
|  |  |  |  |  |  |  |  |  |  |  |  |
| Monica L. Bertoia | 2014 | Cohort study | Netherlands | Women: 143 | 424 | Hcy | Median(μmol/L) Q1: 10.7 Q2: 13.2 Q3: 16.7 | Model 1: 1 1.12 (0.70-1.78) 1.05 (0.65-1.71) Model 2: 1 1.03 (0.58-1.82)  1.14 (0.61-2.12) Model 3: 1 1.03 (0.58-1.85) 1.01 (0.53-1.93) |  | Model 1: Adjusted for matching factors [age, race (women in the NHS only), month of blood draw, fasting status, and smoking]. Model 2: Model 1 þ triglycerides, HDL-C, LDL-C, hsCRP, HbA1c, cystatin C, pack-years of smoking, hypertension, diabetes, family history of myocardial infarction, BMI, alcohol, and postmenopausal hormone use (women only). Model 3: Model 2 þ dietary intakes of total fiber and B vitamins. |  |
|  |  |  |  | Men: 143 | 428 |  | Median(μmol/L) Q1: 11.7 Q2: 14.3 Q3: 18.6 | Model 1: 1  1.72 (1.05-2.81) 2.40 (1.45-3.98) Model 2: 1 1.44 (0.77-2.68)  2.17 (1.08-4.38) Model 3: 1 1.46 (0.78-2.75) 2.37 (1.16-4.82) |  |  |  |
| Eliseo Guallar | 2005 | Cross-sectional study | US | 310 | 4137 | Hcy | Q1: ≤6.6(μmol/L) Q2: 6.6-7.8 Q3: 7.8–9.1 Q4: 9.1–11.0 Q5:>11.0 | Model 1: 1.00 1.18(0.47, 2.93) 1.21( 0.54, 2.70) 1.39(0.68, 2.85) 1.92( 0.95, 3.88) Model 2: 1.00 1.14( 0.45, 2.92) 1.18(0.50, 2.79) 1.40( 0.60, 3.25) 1.87( 0.79, 4.42) Model 3: 1 1.04(0.40, 2.74) 1.02(0.44, 2.40) 1.11(0.49, 2.53) 1.37(0.58, 3.21) Model 4: 1 0.94(0.36, 2.48) 0.87(0.36, 2.08) 0.87(0.37, 2.04) 0.89(0.35, 2.26) |  | Model 1 adjusted for age, sex, race/ethnicity, and education. Model 2 adjusted for age, sex, race/ethnicity, and education. Model 3 further adjusted for lead and cadmium; lead and cadmium further adjusted for homocysteine. Model 4 further adjusted for smoking (never/former/current and cigarettes day) and estimated glomerular filtration rate. |  |
| Daisy G.M. Bloemenkamp | 2002 | Case-control study | Netherlands | 212 | 475 | Hcy | Q1: ≤1.35mg/l Q2: 1.36-1.58 Q3: 1.59-1.86 Q4: ≥1.87 | Unadjusted: 1 0.9 (0.6–1.5) 0.7 (0.4–1.2) 1.3 (0.9–2.1) Adjusted for Age, Smoking, and Education: 1 0.8 (0.5–1.4) 0.7 (0.4–1.2) 1.2 (0.7–1.9) Multiply Adjusted: 1 0.5 (0.3–0.9) 0.5 (0.3–1.0) 0.8 (0.4–1.4) |  | Adjusted for age, body mass index, smoking, hypercholesterolemia, diabetes mellitus, hypertension, education, oral contraceptive use, C-reactive protein and homocysteine levels, and cumulative number of infections. |  |
| Rachel P. Wildman | 2005 | Cohort study | Non-Hispanic blacks and Mexican Americans | 4787 |  | FIB | Q1: ≤282 mg/dL Q2: 282–333 Q3: 333–376 Q4: >376 |  | 1 0.95(0.48–1.89) 2.26(1.18–4.32) 3.19(1.70–5.98)  1 0.85( 0.41–1.76) 1.87(0.98–3.56) 2.49(1.27–4.85) | Adjusted for Age, Gender, Race and Ethnicity   Adjusted for age, gender, race/ethnicity, education, smoking status, diabetes, physical inactivity, total cholesterol, body mass index, and systolic blood pressure |  |
| Khurram Nasir | 2005 | Cross-sectional study | U.S. | 220 | 3729 | FIB | Median level(g/L) Q1: 2.9 Q2: 3.4 Q3: 3.9 Q4: 4.6 |  | Model 1: 1 0.84 (0.37–1.90) 1.84 (0.77–4.40) 3.40 (1.40–8.29) Model 2: 1 0.74 (0.32–1.73) 1.57 (0.63–3.87) 2.68 (1.03–6.94) Model 3: 1 0.70 (0.30–1.62) 1.42 (0.60–3.37) 2.21 (0.88–5.58) | Model 1: Adjusted for age, gender, and race. Model 2: Further adjusted for smoking status, diabetes, hypertension, high cholesterol, GFR (normal, mild reduction, moderate reduction), and BMI (normal, overweight, obese). Model 3: Further adjusted for all other inflammatory markers |  |
| SHUAI-BING LI | 2013 | Case-control study | Chinese | 145 | 837 | HCY | Q1: <12.52μmol/l Q2: 12.52-15.89 Q3: 15.89-19.26 Q4: >19.26 | Model 1 : 1 2.44 (1.73-3.92) 3.22 (2.46-4.79) 3.49 (1.32-3.70) Model 2: 1 1.87 (0.82-2.65) 2.28 (1.61-5.24) 2.14 (1.07-3.11) Model 3: 1 1.03 (1.14-3.76) 1.17 (2.12-4.55) 1.15 (0.49-2.69) |  | Model 1, adjusted for age and gender; model 2, further adjusted for smoking status, diabetes, hypertension, high cholesterol, use of medication and body mass index (normal, overweight or obese); model 3, further adjusted for all other inflammatory markers |  |
|  |  |  |  |  |  | FIB | Q1: <276mg/dl Q2: 276-357 Q3: 357-438 Q4: >438 | Model 1 : 1 0.89 (0.87-2.90) 2.34 (1.77-4.40) 2.35 (1.52-5.29) Model 2: 1 0.65 (0.62-2.73) 1.97 (0.63-3.87) 1.93 (1.02-4.01)  Model 3: 1 0.50 (0.36-1.42) 1.42 (0.82-3.37) 1.21 (0.88-5.57) |  | Model 1, adjusted for age and gender; model 2, further adjusted for smoking status, diabetes, hypertension, high cholesterol, use of medication and body mass index (normal, overweight or obese); model 4, further adjusted for all other inflammatory markers |  |
| Nketi I. Forbang | 2016 | Cohort study | European American 36%, AA 29%, Hispanic American 23%, Chinese American12%. |  |  | LPa | Q1: ≤8mg/dl Q2: 8-18 Q3:18-40 Q4:≥40 | 1 1.22(0.91-1.65) 1.57(1.18-2.09) 1.89(1.44-2.50) | 1 1.13 (0.83-1.54)  1.21 (0.89-1.66)  1.28 (0.93-1.75) | Model adjusted for age, sex, ethnicity, hypertension, diabetes, smoking, total and high-density lipoprotein (HDL) cholesterol, fibrinogen, ln(interleukin-6), ln(D-dimer), and ln(homocysteine). |  |
| Monica L. Bertoia | 2013 | Case-control study | Korea | women: 144 | 432 | LPa | Q1: 0.1-8.4mg/dL Q2: 8.5-35.0  Q3: 35.1-144.6 |  | Model 1: 1 1.04 (0.62-1.75)  2.64 (1.64-4.26)  Model 2: 1 1.11 (0.64-1.94) 2.76 (1.64-4.67) Model 3: 1 1.37 (0.72-2.59)  2.91 (1.58-5.36) |  |  |
|  |  |  |  | men: 143 | 429 | LPa | Q1: 0.1-2.4mg/dL Q2: 2.5-12.0  Q3: 12.1-129.1 |  | Model 1: 1 1.88 (1.13-3.13)  2.51 (1.49-4.21)  Model 2: 1 1.27 (0.73-2.22)  1.91 (1.08-3.39)  Model 3: 1 1.20 (0.64-2.25) 1.59 (0.84-3.00) |  |  |
| Deepti Gurdasani | 2012 | Cohort study | UK | 596 | 212981 | LPa | Q1: 0.1–6.2mg/dl Q2: 5.9–11.7 Q3: 11.2–27.9 Q4: 26.6–175.0 |  | Model 1: 1 1.01(0.79–1.31)  1.04(0.81–1.34) 1.94(1.55–2.43) Model 2: 1 1.07(0.82–1.41) 1.06(0.81–1.38) 2.09(1.64–2.65) Model 3: 1 1.02(0.76–1.37) 1.09(0.82–1.45) 2.06(1.59–2.67) | Model 1 adjusted for age and sex. Model 2 adjusted for age, sex, body mass index, total cholesterol adjusted for Lp(a) levels, HDL cholesterol, and triglycerides. Model 3 was adjusted for all covariates in Model 2 in addition to smoking, alcohol consumption, baseline antihypertensive therapy, baseline lipid-lowering therapy, diabetes mellitus, physical activity level, serum creatinine levels, fibrinogen levels, apolipoprotein A-I, apolipoprotein B, CRP levels, history of myocardial infarction at baseline, history of stroke at baseline, family history of myocardial infarction, family history of stroke, postmenopausal status, and use of hormone replacement therapy |  |
| Stefano Volpato | 2010 | Cohort study | Italy | 1002 |  | LPa | Q1: 0–3.5mg/dl Q2: 3.6–12.3 Q3: 12.4–32.8 Q4: 32.9–175.9 | Model 1: 1 1.33 (0.73–2.41) 1.34 (0.73–2.46)  2.05 (1.16–3.62) Model 2:  1 1.32 (0.71–2.44) 1.33 (0.72–2.47) 2.00 (1.11–3.61) Model 3: 1 1.27 (0.68–2.38)  1.25 (0.67–2.34) 1.83 (1.01–3.33) |  | Model 1 was adjusted for age, gender, smoking, and alcohol intake; model 2 had additional adjustment for body mass index, lipid parameters (low-density lipoprotein cholesterol, high-density lipoprotein cholesterol), diabetes, and hypertension; model 3 also was adjustment for inflammation score. |  |
| Aruna D. Pradhan | 2008 | Cohort study | US | 100 | 27835 | FIB | Q1: <332.1mg/dl Q2: 332.1-433.6 Q3: >433.6 | Age-adjusted HR: 1 1.1 (0.6–2.1) 1.9 (1.1–3.2) | 1 1.1 (0.6–2.0) 1.4 (0.8–2.5) | Adjusted for age, smoking, history of diabetes, history of hypertension, menopausal HT, and BMI. |  |
|  |  |  |  |  |  | Hcy | Q1: <9.7 μmol/l Q2: 9.7-14.5 Q3: >14.5 | 1 1.8 (1.0–3.2) 1.9 (1.1–3.3) | 1 1.5 (0.9–2.7)  1.3 (0.7–2.3) | Adjusted for age, smoking, history of diabetes, history of hypertension, menopausal HT, and BMI. |  |
|  |  |  |  |  |  | LPa | Q1: <5.8mg/dl Q2: 5.8-51.4 Q3: >51.4 | 1 1.1 (0.6–1.8)  1.6 (1.0–2.6) | 1 1.1 (0.6–1.9)  1.6 (1.0–2.6) | Adjusted for age, smoking, history of diabetes, history of hypertension, menopausal HT, and BMI. |  |
| Benjamin Dieplinger | 2007 | Case-control study | Linz, Austria | 213 | 213 | LPa | Q1: <22mg/L Q2: 22-60 Q3: 60-195 Q4: >195 | 1 1.01 (0.64–1.88) 1.02 (0.59–1.75)  2.42 (1.39–4.19) |  |  |  |
| Paul M. Ridker | 2001 | Case-control study | US | 140 | 140 | HCY | Q1: <1.20mg/l Q2: 1.20-1.49 Q3: 1.49-1.68 Q4: >1.68 | 1 1.0 (0.5-1.9) 1.2 (0.6-2.3) 1.0 (0.5-1.9) | 1 1.0 (0.5-2.1) 1.3 (0.7-2.6) 1.1 (0.5-2.1) |  |  |
|  |  |  |  |  |  | FIB | Q1: <0.30g/dl Q2: 0.30-0.34 Q3: 0.34-0.40 Q5: >0.40 | 1 1.2 (0.6-2.6) 1.5 (0.8-3.2) 2.3 (1.2-4.7) | 1 1.2 (0.6-2.5) 1.4 (0.7-2.9) 2.2 (1.1-4.7) |  |  |
