# Supplementary material for: Relationship between serum homocysteine, fibrinogen, lipoprotein-a level, and peripheral arterial disease: a dose–response meta-analysis
Source: Eur J Med Res. 2022 Nov 21;27:261. doi: 10.1186/s40001-022-00870-1 (PMC9677707; doi:10.1186/s40001-022-00870-1)

**Supplementary Figure 1**. Weighted mean difference (WMDs) and 95% confidence intervals (CIs) of the selected studies and the pooled Hcy levels (A), FIB level (B) and LPa level (C) in patients with PAD and control subjects.


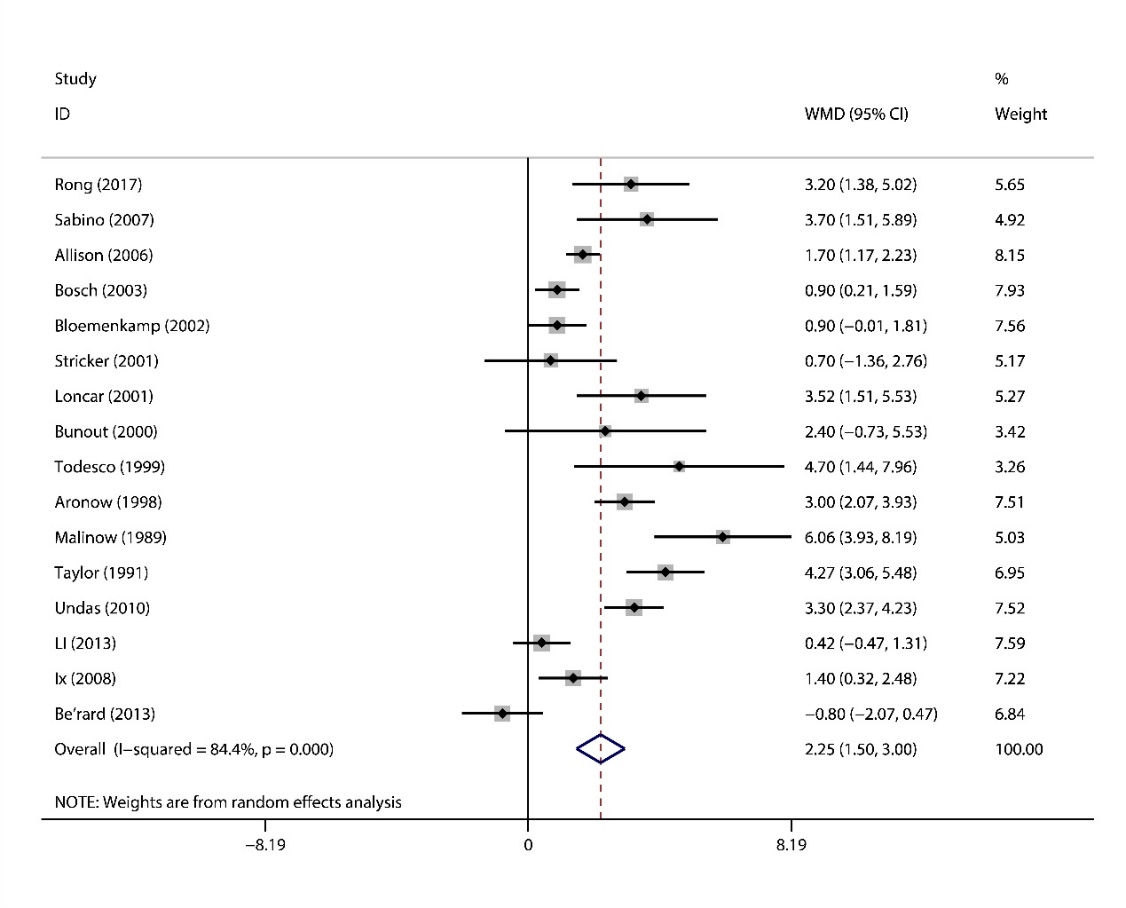


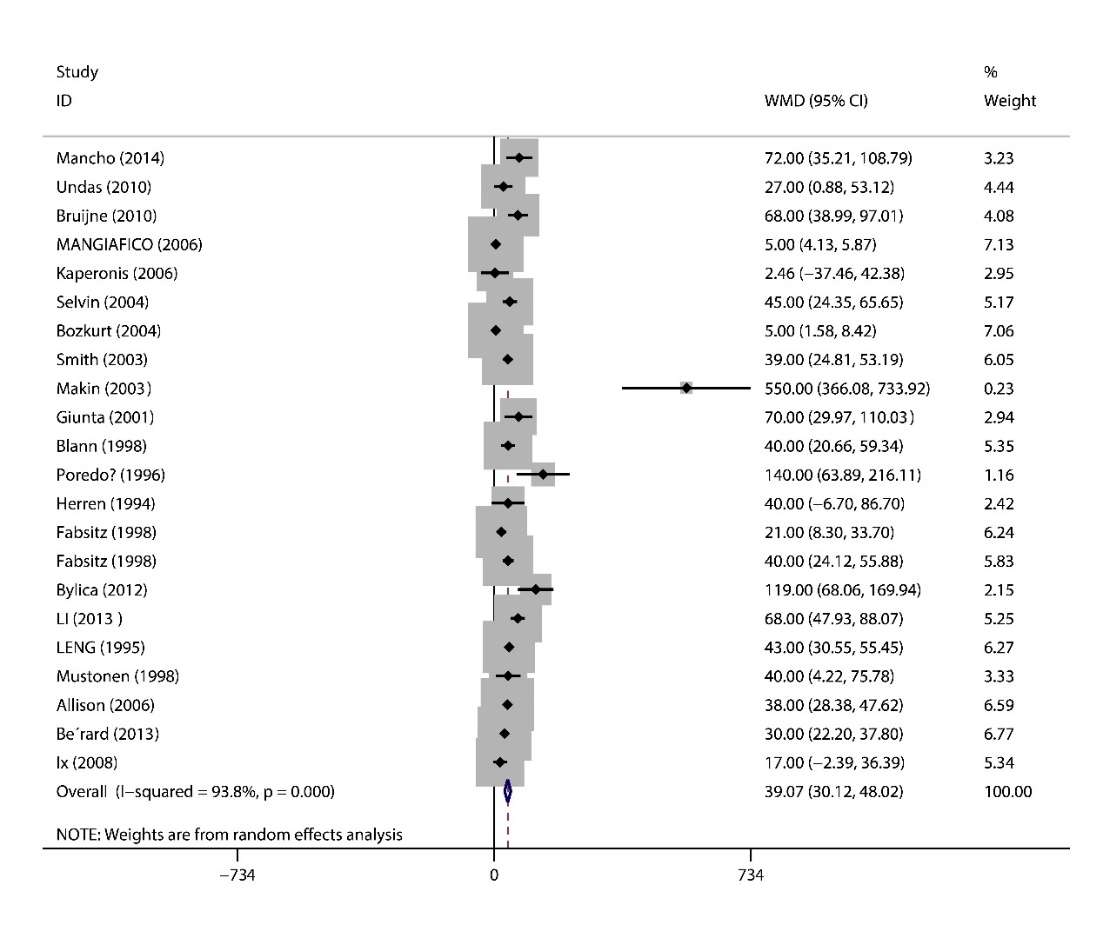


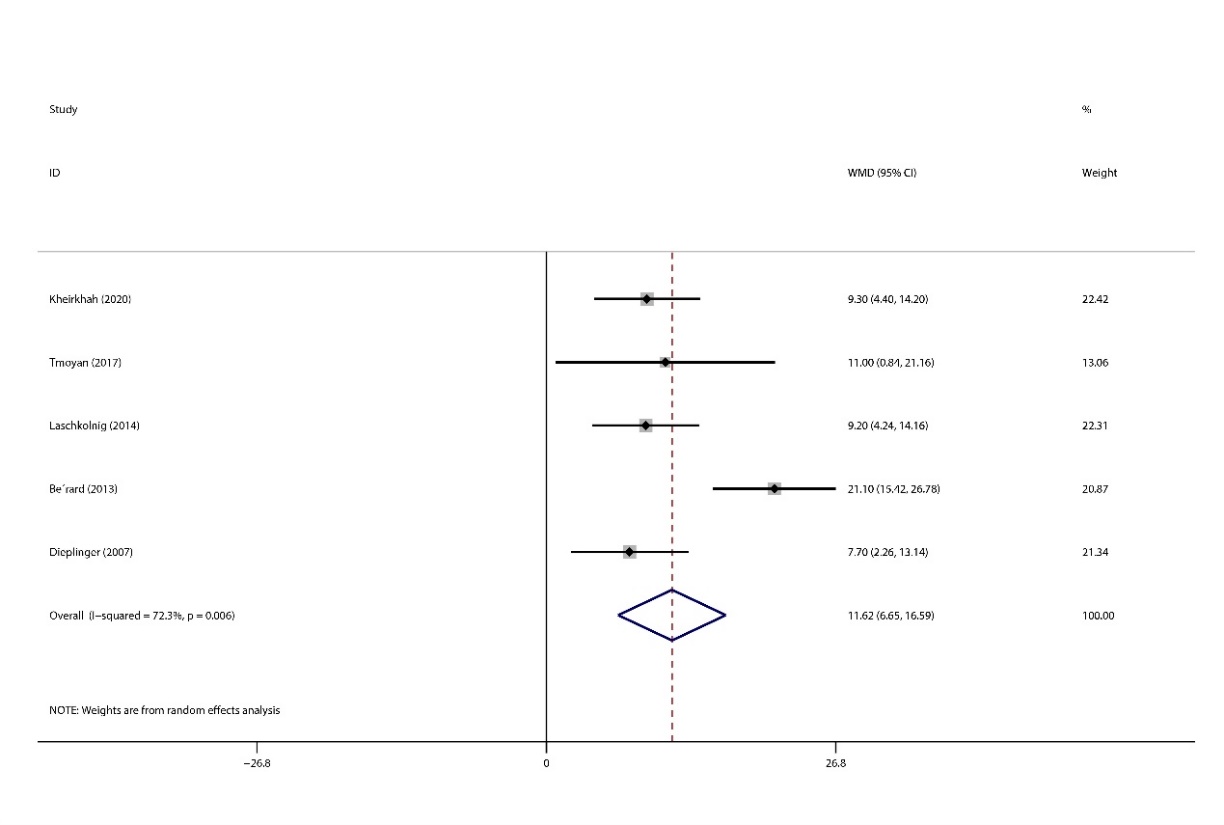

Supplement: Supplementary file 3 — Additional file 3: Figure S1. Weighted mean difference (WMDs) and 95% confidence intervals (CIs) of the selected studies and the pooled Hcy levels (A), FIB level (B) and LPa level C in patients with PAD and control subjects. [file 40001_2022_870_MOESM3_ESM.docx]
