# Supplementary material for: Relationship between serum homocysteine, fibrinogen, lipoprotein-a level, and peripheral arterial disease: a dose–response meta-analysis
Source: Eur J Med Res. 2022 Nov 21;27:261. doi: 10.1186/s40001-022-00870-1 (PMC9677707; doi:10.1186/s40001-022-00870-1)
Supplement: Supplementary file 4 — Additional file 4: Table S3. Table of relative risks (95% confidence intervals) from the nonlinear dose–response analysis of Hcy, FIB, LPa and risk of PAD. [file 40001_2022_870_MOESM4_ESM.docx]

**Supplementary Table 3.** Table of relative risks (95% confidence intervals) from the nonlinear dose-response analysis of Hcy, FIB, LPa and risk of PAD.

| Hcy | | | | | | | |
| --- | --- | --- | --- | --- | --- | --- | --- |
| Model | Non-linear | | | | | | |
| Center value | 9.07 | | | | | | |
| Num knots | 3 | | | | | | |
| Knot values | 7, 9, 12 | | | | | | |
| Num obs | 26 | | | | | | |
| Num studies | 7 | | | | | | |
| R-squared | 0.18 | | | | | | |
| Root MSE | 0.28 | | | | | | |
|  | Coef. | Robust Std. Err. | t | P>\|t\| | [95% Conf. Interval] | |  |
| _doses1 | -0.4353112 | 0.4764703 | -0.91 | 0.396 | -1.601192 | 0.7305695 |  |
| _doses2 | 0.418017 | 0.4253958 | 0.98 | 0.364 | -0.6228892 | 1.458923 |  |
| _cons | 3.79536 | 4.169266 | 0.91 | 0.398 | -6.406465 | 13.99719 |  |
| Levels of Hcy | RR | 95% CI |  |  |  |  |  |
| 9.0699997 | 1 | (0.99-1.00) |  |  |  |  |  |
| 10.27 | 0.86 | (0.60-1.25) |  |  |  |  |  |
| 10.77 | 0.86 | (0.57-1.29) |  |  |  |  |  |
| 11.17 | 0.87 | (0.58-1.31) |  |  |  |  |  |
| 11.52 | 0.89 | (0.59-1.32) |  |  |  |  |  |
| 11.57 | 0.89 | (0.60-1.32) |  |  |  |  |  |
| 11.67 | 0.89 | (0.61-1.32) |  |  |  |  |  |
| 12.469999 | 0.94 | (0.67-1.32) |  |  |  |  |  |
| 12.715 | 0.96 | (0.69-1.33) |  |  |  |  |  |
| 12.92 | 0.97 | (0.71-1.33) |  |  |  |  |  |
| 13.12 | 0.98 | (0.73-1.33) |  |  |  |  |  |
| 13.87 | 1.03 | (0.80-1.34) |  |  |  |  |  |
| 14.320001 | 1.07 | (0.85-1.34) |  |  |  |  |  |
| 14.715 | 1.09 | (0.89-1.35) |  |  |  |  |  |
| 15.02 | 1.12 | (0.92-1.36) |  |  |  |  |  |
| 15.070001 | 1.12 | (0.92-1.36) |  |  |  |  |  |
| 15.849999 | 1.18 | (1.00-1.39) |  |  |  |  |  |
| 15.97 | 1.19 | (1.01-1.40) |  |  |  |  |  |
| 18.67 | 1.42 | (1.17-1.72) |  |  |  |  |  |
| 19.220001 | 1.47 | (1.18-1.83) |  |  |  |  |  |
| We took the doses of 11.7 to 18.6 μmol/l at the first spline which corresponds to 2.84072 to 11.12 at the second spline to estimate an overall linear trend per 1μmol/l of serum Hcy concentration increase as follows:  ${OR}_{trend}$=exp$\left[ \frac{\left( 18.6-11.7 \right)\times\beta_{1}+\left( 11.12-2.84 \right)\times\beta_{2}}{18.6-11.7} \right]=1.07$ | | | | | | | |
| FIB | | | | | | | |
| Model | Non-linear | | | | | | |
| Center value | 268.67999 | | | | | | |
| Num knots | 3 | | | | | | |
| Knot values | 268, 318, 370 | | | | | | |
| Num obs | 19 | | | | | | |
| Num studies | 5 | | | | | | |
| R-squared | 0.28 | | | | | | |
| Root MSE | 0.35 | | | | | | |
|  | Coef. | Robust Std. Err. | t | P>\|t\| | [95% Conf. Interval] | |  |
| _doses1 | 0.0005478 | 0.0036163 | 0.15 | 0.887 | -0.0094927 | 0.0105883 |  |
| _doses2 | 0.0020499 | 0.0027628 | 0.74 | 0.499 | -0.0056209 | 0.0097208 |  |
| _cons | -0.1553034 | 0.9804146 | -0.16 | 0.882 | -2.877371 | 2.566764 |  |
| Levels of FIB | RR | 95% CI |  |  |  |  |  |
| 268.67999 | 0.99 | (0.96-1.02) |  |  |  |  |  |
| 308.67999 | 1.03 | (0.81-1.30) |  |  |  |  |  |
| 318.67999 | 1.05 | (0.80-1.37) |  |  |  |  |  |
| 319.67999 | 1.05 | (0.80-1.38) |  |  |  |  |  |
| 349.67999 | 1.14 | (0.83-1.57) |  |  |  |  |  |
| 358.67999 | 1.18 | (0.85-1.62) |  |  |  |  |  |
| 366.67999 | 1.21 | (0.88-1.67) |  |  |  |  |  |
| 368.67999 | 1.22 | (0.88-1.68) |  |  |  |  |  |
| 370.07999 | 1.22 | (0.89-1.69) |  |  |  |  |  |
| 409.67999 | 1.41 | (1.01-1.97) |  |  |  |  |  |
| 418.67999 | 1.46 | (1.03-2.05) |  |  |  |  |  |
| 430.67999 | 1.52 | (1.07-2.16) |  |  |  |  |  |
| 438.67999 | 1.56 | (1.09-2.24) |  |  |  |  |  |
| 471.57999 | 1.76 | (1.18-2.62) |  |  |  |  |  |
| 511.67999 | 2.03 | (1.28-3.21) |  |  |  |  |  |
|  |  |  |  |  |  |  |  |
|  |  |  |  |  |  |  |  |
|  |  |  |  |  |  |  |  |
|  |  |  |  |  |  |  |  |
|  |  |  |  |  |  |  |  |
| A RCS was created (with three knots at 268, 318 and 270 across the increment distribution), which generates two splines, and these were then employed for the potential nonlinear dose-specific modeling. The regression parameter estimates of the first spline and the second spline were (β1)and (β2) respectively. The overall linear trend can be estimated by comparing the change in OR between an increment of FIB level of 320 and 484.3 mg/dl (spline 1) (corresponding to 13.5 and 243.58 at the second spline); the overall linear trend (per 10mg/dl increase) then would be  exp$\left[ \frac{\left( 484.3-320 \right)\times\beta_{1}+\left( 243.58-13.5 \right)\times\beta_{2}}{\left( 484.3-320 \right)}\times10 \right]=1.03$ | | | | | | | |
| LPa | | | | | | | |
| Model | Non-linear | | | | | | |
| Center value | 2.72 | | | | | | |
| Num knots | 3 | | | | | | |
| Knot values | 2, 19, 74 | | | | | | |
| Num obs | 21 | | | | | | |
| Num studies | 6 | | | | | | |
| R-squared | 0.9 | | | | | | |
| Root MSE | 0.09 | | | | | | |
|  | Coef. | Robust Std. Err. | t | P>\|t\| | [95% Conf. Interval] | |  |
| _doses1 | 0.007384 | 0.0017644 | 4.18 | 0.009 | 0.0028484 | 0.0119195 |  |
| _doses2 | -0.002629 | 0.0036084 | -0.73 | 0.499 | -0.011905 | 0.0066465 |  |
| _cons | -0.008472 | 0.0225447 | -0.38 | 0.722 | -0.066425 | 0.0494812 |  |
| Levels of LPa | RR | 95% CI |  |  |  |  |  |
| 2.72 | 1.01 | (0.97-1.05) |  |  |  |  |  |
| 8.3699999 | 1.05 | (1.02-1.09) |  |  |  |  |  |
| 8.7200003 | 1.06 | (1.03-1.09) |  |  |  |  |  |
| 8.9200001 | 1.06 | (1.03-1.09) |  |  |  |  |  |
| 12.72 | 1.09 | (1.06-1.12) |  |  |  |  |  |
| 19.119999 | 1.14 | (1.10-1.18) |  |  |  |  |  |
| 20.219999 | 1.15 | (1.10-1.20) |  |  |  |  |  |
| 23.57 | 1.17 | (1.12-1.23) |  |  |  |  |  |
| 28.42 | 1.21 | (1.15-1.28) |  |  |  |  |  |
| 28.719999 | 1.22 | (1.16-1.28) |  |  |  |  |  |
| 50.720001 | 1.39 | (1.31-1.48) |  |  |  |  |  |
| 74.019997 | 1.59 | (1.46-1.72) |  |  |  |  |  |
| 100.37 | 1.84 | (1.61-2.09) |  |  |  |  |  |
| 105.37 | 1.89 | (1.64-2.17) |  |  |  |  |  |
| 142.67 | 2.33 | (1.86-2.91) |  |  |  |  |  |
| 178.17 | 2.83 | (2.09-3.85) |  |  |  |  |  |
| There were three knots (at 2, 19 and 74) using RCSs. Estimated regression parameters were 0.007384 for β1 and -0.002629 for β2. Given the linear relationship, the linear trend (for per 10 mg/dl increase) between 29 mg/dl and 179.7 mg/dl (corresponding to 3.54 and 104.86 at the second spline) would be  exp$\left[ \frac{\left( 179.7-29 \right)\times\beta_{1}+\left( 104.86-3.54 \right)\times\beta_{2}}{179.7-29}\times10 \right]=1.06$  thus, the risk of PAD increases by 6% for per 10 mg/dl serum LPa increase. | | | | | | | |
