# Supplementary material for: Relationship between serum homocysteine, fibrinogen, lipoprotein-a level, and peripheral arterial disease: a dose–response meta-analysis
Source: Eur J Med Res. 2022 Nov 21;27:261. doi: 10.1186/s40001-022-00870-1 (PMC9677707; doi:10.1186/s40001-022-00870-1)
Supplement: Supplementary file 5 — Additional file 5: Table S4. Table of relative risks (95% confidence intervals) from the linear dose–response analysis of Hcy, FIB, LPa and risk of PAD. [file 40001_2022_870_MOESM5_ESM.docx]

**Supplementary Table 4.** Table of relative risks (95% confidence intervals) from the linear dose-response analysis of Hcy, FIB, LPa and risk of PAD.

| Levels of Hcy | exp(xb) | (95% CI) | Levels of FIB | exp(xb) | (95% CI) | Levels of LPa | exp(xb) | (95% CI) |
| --- | --- | --- | --- | --- | --- | --- | --- | --- |
| 9.0699997 | 0.92 | (0.78-1.09) | 268.67999 | 0.95 | (0.87-1.05) | 2.72 | 1.02 | (0.98-1.07) |
| 10.27 | 0.96 | (0.81-1.13) | 308.67999 | 1.07 | (0.94-1.22) | 8.3699999 | 1.06 | (1.02-1.10) |
| 10.77 | 0.97 | (0.82-1.15) | 318.67999 | 1.1 | (0.95-1.27) | 8.7200003 | 1.06 | (1.02-1.10) |
| 11.17 | 0.99 | (0.83-1.17) | 319.67999 | 1.1 | (0.95-1.27) | 8.9200001 | 1.06 | (1.03-1.10) |
| 11.52 | 1 | (0.84-1.19) | 349.67999 | 1.2 | (0.99-1.45) | 12.72 | 1.09 | (1.06-1.12) |
| 11.57 | 1 | (0.84-1.19) | 358.67999 | 1.23 | (1.00-1.51) | 19.119999 | 1.13 | (1.11-1.15) |
| 11.67 | 1 | (0.84-1.19) | 366.67999 | 1.26 | (1.01-1.57) | 20.219999 | 1.14 | (1.12-1.16) |
| 12.469999 | 1.03 | (0.86-1.23) | 368.67999 | 1.26 | (1.01-1.58) | 23.57 | 1.16 | (1.14-1.18) |
| 12.715 | 1.04 | (0.86-1.24) | 370.07999 | 1.27 | (1.01-1.59) | 28.42 | 1.19 | (1.18-1.21) |
| 12.92 | 1.04 | (0.87-1.25) | 409.67999 | 1.42 | (1.05-1.91) | 28.719999 | 1.2 | (1.18-1.21) |
| 13.12 | 1.05 | (0.87-1.26) | 418.67999 | 1.45 | (1.06-1.99) | 50.720001 | 1.36 | (1.31-1.43) |
| 13.87 | 1.08 | (0.89-1.30) | 430.67999 | 1.5 | (1.07-2.11) | 74.019997 | 1.57 | (1.44-1.71) |
| 14.320001 | 1.09 | (0.90-1.33) | 438.67999 | 1.54 | (1.08-2.19) | 100.37 | 1.84 | (1.62-2.09) |
| 14.715 | 1.11 | (0.90-1.35) | 471.57999 | 1.69 | (1.12-2.55) | 105.37 | 1.89 | (1.65-2.17) |
| 15.02 | 1.12 | (0.91-1.37) | 511.67999 | 1.89 | (1.16-3.08) | 142.67 | 2.37 | (1.93-2.90) |
| 15.070001 | 1.12 | (0.91-1.37) |  |  |  | 178.17 | 2.93 | (2.25-3.81) |
| 15.849999 | 1.15 | (0.93-1.42) |  |  |  |  |  |  |
| 15.97 | 1.15 | (0.93-1.43) |  |  |  |  |  |  |
| 18.67 | 1.26 | (0.98-1.61) |  |  |  |  |  |  |
| 19.220001 | 1.28 | (0.99-1.65) |  |  |  |  |  |  |
| For linear trends based on dose, the trend in OR per 1μmol/l serum Hcy increase was:  ${OR}_{trend}$=exp$\left[ \frac{\left( 18.67-11.17 \right)\times\beta_{1}}{18.67-11.17} \right]=1.033$ | | | Computing a linear trend revealed a % increase in the risk of PAD for every 10mg/dl increase of FIB concentration.  ${OR}_{trend}$=exp$\left[ \frac{\left( 460-281.4 \right)\times\beta_{1}}{460-281.4}\times10 \right]=1.03$ | | | The model was run without spline (using the linear dose fit), the trend was 6% increase in the risk of PAD per 10mg/dl increase of LPa concentration.  ${OR}_{trend}$=exp$\left[ \frac{\left( 179.7-21.75 \right)\times\beta_{1}}{179.7-21.75}\times10 \right]=1.06$ | | |
